# Age-Driven Lipid Remodeling Activates Lysosome-Mediated Plasma Membrane Repair

**DOI:** 10.21203/rs.3.rs-8607320/v1

**Published:** 2026-01-20

**Authors:** Emily Tom, Fangyuan Gao, Carolina N. Franco, Adrian Wong, Nathan Kemmerer, Zichen Wang, Qianlan Xu, Yinyin Zhuang, Samuel W. Du, Grazyna Palczewska, Krzysztof Palczewski, Itay Budin, Xiaoyu Shi, Vera L. Bonilha, Johannes Schöeneberg, Karl J. Wahlin, Lauren V. Albrecht, Dorota Skowronska-Krawczyk

**Affiliations:** 1 Department of Physiology and Biophysics, School of Medicine, University of California, Irvine, Irvine, CA; 2 Gavin Herbert Eye Institute – Brunson Center for Translational Vision Research, Department of Ophthalmology and Visual Sciences, University of California, Irvine, Irvine, CA; 3 Department of Pharmaceutical Sciences, School of Pharmacy and Pharmaceutical Sciences, University of California, Irvine, Irvine, CA; 4 Department of Chemistry and Biochemistry, University of California San Diego, La Jolla, CA; 5 Shiley Eye Institute, University of California, San Diego, La Jolla, CA; 6 Department of Pharmacology, University of California San Diego, La Jolla, CA; 7 Department of Developmental and Cell Biology, University of California, Irvine, Irvine, CA; 8 Department of Molecular Biology and Biochemistry, University of California, Irvine, Irvine, CA; 9 Department of Chemistry, University of California, Irvine, Irvine, CA; 10 Department of Biomedical Engineering, University of California, Irvine, Irvine, CA; 11 Department of Ophthalmic Research, Cleveland Clinic, Cleveland, OH; 12 Department of Molecular Medicine, Cleveland Clinic Lerner College of Medicine, School of Medicine, Case Western Reserve University, Cleveland, OH

**Keywords:** plasma membrane repair, aging, lysosomes

## Abstract

The abundance and stoichiometry of membrane lipid species vary across a cell’s lifespan and metabolic state. In the retinal pigment epithelium (RPE), age-related alterations in lipid composition contribute to vision loss and diseases such as age-related macular degeneration (AMD), yet the molecular drivers of these changes remain unclear. Here, we show that age-dependent remodeling of the composition and biophysical properties of the plasma membrane compromises membrane integrity and function. Remarkably, rather than undergoing cell death, affected cells activate a lysosome-dependent plasma membrane repair program to preserve barrier integrity. While this adaptive response may protect RPE structure under metabolic stress, it also drives spatially polarized release of lysosomal contents that potentially can contribute to extracellular matrix remodeling and sub-RPE deposit formation during aging and AMD. Finally, we demonstrate that supplementation with the direct product of the aging-associated lipid elongase ELOVL2 alleviates these phenotypes, providing direct evidence for a critical role of ELOVL2-mediated PUFA elongation in healthy aging. Taken together, our results propose a model in which age-dependent decline in PUFA elongation disrupts the balance between membrane flexibility and stability, initiating a compensatory cycle of membrane stress and repair.

## INTRODUCTION

Aging is associated with widespread remodeling of cellular membranes, yet the molecular drivers and functional consequences of these changes have remained to be understood. The retinal pigment epithelium (RPE) forms a highly specialized monolayer that, in addition to maintaining outer blood-retina barrier integrity, supports photoreceptor health and visual function through active membrane trafficking, nutrient exchange, and daily phagocytosis of photoreceptor outer segments^1^. These processes require precise control of plasma membrane composition, curvature, and repair. With age, the RPE undergoes progressive metabolic and structural decline, characterized by altered lipid handling, impaired lysosomal clearance, and the accumulation of extracellular debris—features that contribute to both physiological aging and the pathogenesis of age-related macular degeneration (AMD)^2,3^. However, the molecular mechanisms linking age-associated metabolic dysfunction to RPE membrane remodeling remain poorly defined.

Cellular membranes are dynamic structures whose lipid composition and biophysical properties are critical for cellular function, including vesicular trafficking, signaling, and cell survival^4,5^. Maintaining membrane integrity is especially critical in post-mitotic RPE cells, which must withstand lifelong mechanical, metabolic, and oxidative stress^6^. Accordingly, disruption of lipid metabolism represents a major vulnerability for RPE membrane homeostasis and the membrane-dependent processes it supports. Polyunsaturated fatty acids (PUFAs), containing more than one double bond, such as docosahexaenoic acid (DHA, 22:6n-3), confer high flexibility to lipid bilayers, where they help sustain the membrane fluidity, curvature and diffusion properties required for phagocytosis, trafficking, and barrier integrity. The enzyme ELOVL2 (Elongation of Very Long-Chain Fatty Acids-Like 2) is a key regulator of PUFA elongation and is essential for the biosynthesis of long-chain (LC-) and very-long-chain (VLC-) PUFAs, including DHA. Importantly, the *ELOVL2* gene has emerged as a robust molecular biomarker of aging, with a progressive increase in methylation levels of its promoter region correlating strongly with chronological age^7-9^. This epigenetic silencing is associated with decreased *ELOVL2* expression in the mouse retina, altered lipid profiles and premature visual decline^10,11^. We recently reported that intravitreal supplementation with tetracosapentaenoic acid (24:5n-3), the direct product of ELOVL2, resulted in improved visual function in aged (18-month-old) mice^11^. These findings suggest that age-related changes in PUFA metabolism may contribute to the functional and structural membrane alterations in the aging retina. Importantly, aging is accompanied by broad shifts in cellular lipid composition, including changes in PUFA abundance, sphingolipid content, and phospholipid class balance, which alter membrane biophysical properties^12-14^ and perturb essential membrane-dependent cellular processes^15^, though a mechanistic understanding remains enigmatic.

Given the central importance of sustaining PUFA levels for membrane stability, we hypothesized that impaired ELOVL2 activity is a key metabolic driver of age-related structural and functional remodeling in the RPE. To test this hypothesis, we integrated transcriptomic, lipidomic, and cellular analyses from human aging RPE, ELOVL2-deficient mouse models, and *in vitro* RPE systems, including RPE differentiated from induced pluripotent stem cells (iPSC-RPE). Our results demonstrate that aging and the loss of ELOVL2 converge on membrane-associated pathways, leading to altered plasma membrane composition, ceramide accumulation, and activation of lysosome-mediated membrane repair responses. These findings reveal that metabolic dysregulation of PUFA elongation alters membrane lipid metabolism and triggers adaptive remodeling of membrane architecture and organellar trafficking, offering new insight into how metabolic aging disrupts plasma membrane homeostasis.

## RESULTS

### Transcriptomic profiling reveals age-related membrane remodeling in the human and mouse RPE

**Fig 1.**
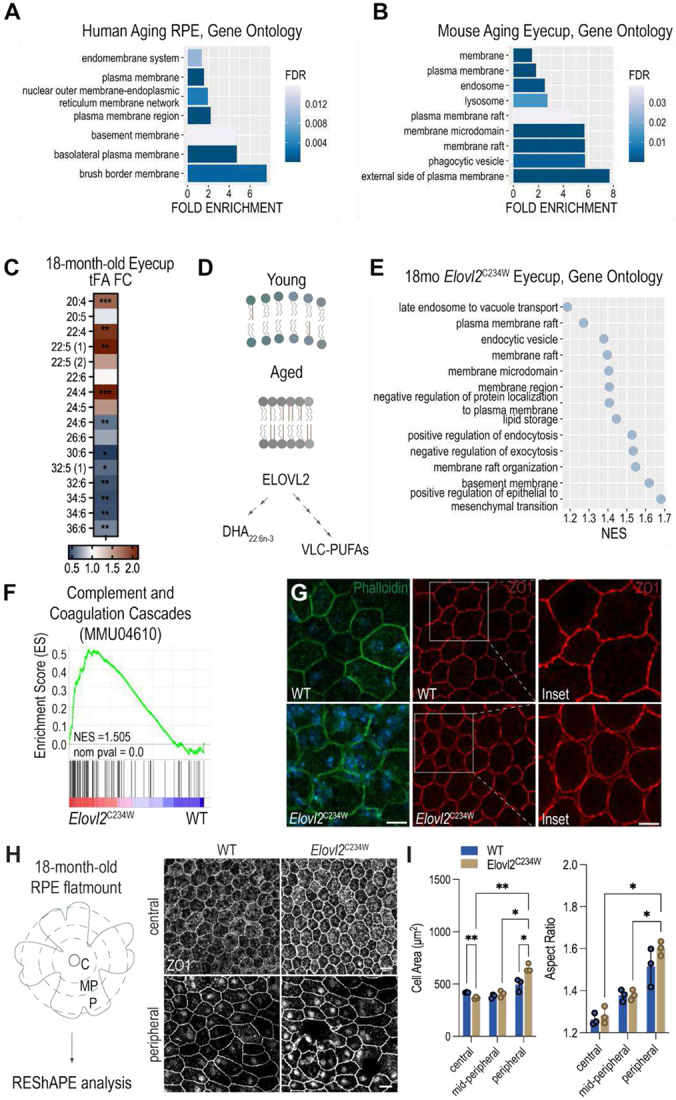
ELOVL2 depletion recapitulates membrane remodeling during aging. **a-b.** Cellular Component overrepresentation analysis of significantly correlated genes (SCGs) and differentially expressed genes (DEGs) from human aging RPE^16^ (**a**) and 18-month-old wild-type mouse eyecups (**b**), respectively. **c.** Heatmap of total fatty acid changes in eyecups from 18-month-old wild-type mice relative to 3-month-old wild-type (*n* = 5 per group). **d.** Schematic illustration of the role of ELOVL2 in the synthesis of LC- and VLC-PUFAs and how loss of activity alters membrane lipid composition during aging. **e.** Gene set enrichment analysis (GSEA) of eyecups from 18-month-old *Elovl2*^C234W^ mutant mice using the GO Cellular Component ontology. **f.** GSEA enrichment plot for the “Complement and Coagulation Cascades” term (KEGG mmu04610) in eyecups from 18-month-old *Elovl2*^C234W^ mutant mice. **g.** Representative confocal images of RPE flatmounts of 18-month-old wild-type and *Elovl2*^C234W^ mice stained with phalloidin (green) and ZO-1 (red). Scale = 20 μm, inset scale = 10 μm. **h.** Left: schematic diagram of central, mid-peripheral and peripheral RPE regions sampled for quantitative morphometry. Right: representative images of RPE flatmounts from central and peripheral regions from 18-month-old wild-type and *Elovl2*^C234W^ mice. Scale = 20 μm. **i.** Quantitative morphometric analyses performed using ReSHAPE to measure RPE cell area and aspect ratio (*n* = 3 animals per group). Data are presented as mean ± SEM. Statistical significance was determined using two-way ANOVA with Geisser-Greenhouse correction (**h**). *P < 0.05, **P < 0.01, ***P < 0.001, and ****P < 0.0001. Source numerical data are available in Source Data.

To identify cellular components most affected by aging in the RPE, we first analyzed transcriptomic data from a previously published study of healthy aging human RPE donors aged 31 to 93 years^16^. From this dataset, we extracted significantly correlated genes (SCGs), exhibiting a significant positive or negative correlation with age (Benjamini-Hochberg adjusted *P* < 0.05 and ∣β_age_∣ > 0.025). We then performed overrepresentation analysis on the downregulated SCGs using the PANTHER classification system, focusing on the Gene Ontology (GO) “Cellular Component” category^17^. This analysis revealed a significant enrichment of genes associated with membrane-related structures, including the endomembrane system (fold enrichment = 1.37, FDR = 9.68E-03), plasma membrane (fold enrichment = 1.58, FDR = 1.06E-08), and brush border membrane (fold enrichment = 7.6, FDR = 1.74E-03) (Fig. 1A).

To determine whether these age-associated transcriptional signatures are conserved in mice, we performed bulk RNA sequencing on RPE-choroid eyecups from young (3-month-old) and aged (18-month-old) wild-type male C57BL/6J mice. Similar to human RPE, aged mouse eyecups exhibited significant enrichment in genes associated with membrane components, including plasma membrane (fold enrichment = 1.81, FDR = 9.87E-13), membrane microdomain (fold enrichment = 5.69, FDR = 9.03E-08), lysosome (fold enrichment = 2.73, FDR = 1.39E-02), and endosome (fold enrichment = 2.52, FDR = 1.11E-03) (Fig. 1B). These findings suggest that membrane-associated processes are particularly impacted during healthy aging in the RPE and may contribute to age-related changes in cellular function.

### ELOVL2 deficiency recapitulates age-associated membrane transcriptional signatures

Given the strong enrichment of membrane-associated pathways in the aging human and mouse RPE transcriptomes, we hypothesized that altered lipid metabolism may be a key upstream driver of these changes. In particular, PUFAs are critical determinants of membrane fluidity and function, and epidemiological studies have linked dietary PUFA status with AMD risk^18,19^. Moreover, prior analyses of postmortem human donor eyes have reported lower concentrations of LC- and VLC-PUFAs in RPE/choroid from aged individuals suggesting that PUFA depletion is associated with RPE dysfunction in aging^20^. We performed total fatty acid profiling of eyecups from young (3-month-old) and aged (18-month-old) wild-type animals, which revealed significantly increased levels of LC- and VLC-PUFAs 20:4, 22:4, 22:5(1), and 24:4 and significantly decreased levels of 24:6, 30:6, 32:5, 32:6, 34:5, 34:6, and 36:6 in aged eyecups (Fig. 1C, S1A). The depleted fatty acid species correspond to products of PUFA elongation pathways downstream of ELOVL2, suggesting that age-associated changes in ELOVL2 activity may underlie the selective loss of membrane-enriched PUFAs in the aging RPE (Fig. 1D). We therefore tested the extent to which ELOVL2 deficiency could recapitulate similar molecular signatures *in vivo*. We analyzed transcriptomic changes in 18-month-old *Elovl2*^*C234W*^ mutant mice compared to age-matched wild-type controls, which carry a point mutation that disrupts the enzyme’s substrate- binding ability^10^. Differential expression analysis was followed by Gene Set Enrichment Analysis (GSEA) using the GO Cellular Component database to identify enriched structural categories^21^. Similar to the transcriptomic changes observed in human aging RPE, *Elovl2*^*C234W*^ mutant eyecups demonstrated a significant enrichment of genes associated with plasma membrane raft (NES = 1.27, *P* < 10E-05), endocytic vesicle (NES = 1.38, *P* < 10E-05), membrane raft organization (NES = 1.55, *P* < 10E-05), and basement membrane (NES = 1.62, *P* < 10E-05) relative to age-matched wild-type animals (Fig. 1E). Furthermore, genes associated with the positive regulation of epithelial to mesenchymal transition (EMT) were significantly enriched (NES = 1.68, *P* < 10E-05), a phenotype previously described in aging RPE and linked to tissue remodeling and functional decline^22^ (Fig. 1E). In addition to these structural and phenotypic changes, we observed significant upregulation of the complement and coagulation cascades pathway (KEGG mmu04610) (NES = 1.51, *P* < 10E-05), consistent with heightened innate immune activation and chronic inflammatory signaling often seen in both aging and AMD retinas^23-25^ (Fig. 1F).

### Morphometric and ultrastructural remodeling of RPE in ELOVL2-deficient mice

To corroborate transcriptomic evidence of structural and phenotypic remodeling, we next performed morphometric analysis using RPE flatmount preparations from 18-month-old wild-type and *Elovl2*^*C234W*^ mutant mice. Eyecups were carefully dissected to preserve the RPE monolayer, and flatmounts were stained for F-actin organization and the tight junction protein, zona occludens-1 (ZO-1). In *Elovl2*^*C234W*^ RPE, both phalloidin and ZO-1 staining highlighted the presence of circular, poorly interdigitated cells embedded within an otherwise hexagonally organized monolayer (Fig. 1G). To capture potential regional variation in RPE morphology, each flatmount was subdivided into central, mid-peripheral, and peripheral zones based on radial distance from the optic nerve head and imaged for quantitative assessment (Fig. 1H). Morphometric analysis was conducted using the REShAPE pipeline, a machine learning-based software that enables high-throughput quantification of RPE cell shape characteristics, such as area, aspect ratio, number of neighboring cells, and hexagonality^26^. Consistent with regionalized structural remodeling, *Elovl2*^*C234W*^ mutant mice exhibited decreased cell area in the central RPE and increased cell area in the peripheral RPE compared to wild-type mice, as well as increases in aspect ratio in both central and peripheral regions, phenotypes previously associated with aging RPE^27^ (Fig. 1I). These aberrant cell shapes indicate disrupted cytoskeletal tension and weakened junctions, suggesting that the loss of ELOVL2 activity drives structural changes in the RPE that mirror aging-associated phenotypes.

Because lipid imbalance and structural remodeling in the RPE can lead to the accumulation of fluorescent byproducts in the retina, we next used two-photon excitation (TPE) coupled with fluorescence lifetime imaging microscopy (FLIM), as previously described^28^, to assess potential fluorophore changes *in vivo*. Using this approach, we detected a multitude of discrete, brightly fluorescent punctate deposits in the sub-retinal space of *Elovl2*^*C234W*^ animals, but not in wild-type animals (Fig. S1B). Fluorescence from these deposits was detected at the 580-680 nm emission spectral range corresponding to retinal condensation products (Fig. S1C). Although the RPE of these pigmented mice contains melanin, which displays spectral and phasor FLIM signatures that overlap with those of diretinoid-pyridiniumethanolamine (A2E)^28^, the localization of the signal to the sub-retinal space of the outer retina indicates that retinal condensation products are the predominant fluorophores. This interpretation is further supported by the TPE fluorescence spectra observed in aging mice^29^.

**Fig 2.**
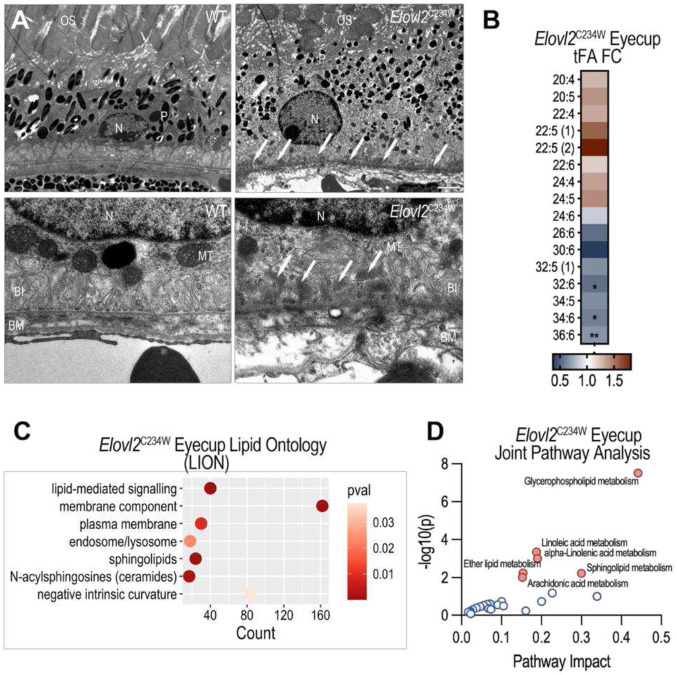
Disrupting LC-PUFA synthesis drives ultrastructural defects and lipid remodeling. **a.** Transmission electron microscopy images of the RPE-photoreceptor interface from 18-month-old wild-type and *Elovl2*^C234W^ mice. OS, outer segments; P, pigment granules; N, nucleus; MT, mitochondria; BI, basal infoldings. Scale = 2 μm (top), 1 μm (bottom). **b.** Heatmap of total fatty acid (tFA) changes in eyecups 18-month-old *Elovl2*^C234W^ mice relative to age-matched wild-type (*n* = 3 per group). **c.** Lipid Ontology (LION) enrichment analysis of significantly altered lipid species in eyecups from 18-month-old *Elovl2*^C234W^ mice relative to age-matched wild-type (*n* = 2 per group). **d.** Joint pathway analysis integrating lipidomics and transcriptomics data from 18-month-old *Elovl2*^C234W^ mice and age-matched wild-type eyecups using MetaboAnalyst. Statistical significance was determined using unpaired t-tests (**b**). *P < 0.05, **P < 0.01, ***P < 0.001, and ****P < 0.0001. Source numerical data are available in Source Data.

To determine how impaired endogenous synthesis of LC-PUFAs affects RPE architecture *in vivo*, we examined ultrastructural changes in 18-month-old wild-type and *Elovl2*^C234W^mutant mice using transmission electron microscopy (TEM) (Fig. 2A). In aged wild-type animals, the RPE maintained well-organized basal infoldings that increase membrane surface area and support metabolic exchange with Bruch’s membrane and the choroid. In contrast, *Elovl2*^C234W^ animals exhibited marked ultrastructural abnormalities. Basal infoldings were substantially decreased or completely lost, resulting in a flattened basal RPE surface and decreased surface area for essential transport and exchange processes^30-32^. Additionally, mutant animals accumulated amorphous sub-RPE material between the RPE basal surface and Bruch’s membrane, resembling early sub-RPE deposits observed during aging and in AMD^33,34^ (Fig. 2A).

### Age- and ELOVL2-associated alterations of the mouse RPE/eyecup lipidome

The phenotypes observed in the RPE of *Elovl2*^C234W^ animals, together with previously published data^10,11^, strongly point to premature aging. Total fatty acid analysis of eyecups from 18-month-old *Elovl2*^C234W^ mice relative to age-matched wild-type controls showed the same overall trend in LC-PUFA depletion as natural aging, indicating a shared pattern of aging- and ELOVL2-mediated PUFA metabolism (Fig. 2B, S2A).

Untargeted global lipidomic profiling showed eyecups from 18-month-old *Elovl2*^C234W^ mutant mice display increased levels of ceramides (Cer), cholesterol esters (ChE), lysophosphatidylcholine (LPC), phosphatidylcholine (PC), phosphatidylethanolamine (PE), and sphingomyelin (SM) and decreased levels of triglycerides (TG) (Fig. S2B, C). To gain insight into the functional implications of these changes, we then performed Lipid Ontology (LION) analysis^35^, which revealed a significant enrichment of lipid species associated with signaling functions and membrane structural components, including those localized to the plasma membrane and endosome/lysosome compartments (Fig. 2C). Additionally, there was a notable accumulation of ceramides known to increase the molecular order (rigidity) of phospholipids in membranes and promote negative intrinsic membrane curvature, a biophysical property that facilitates the formation of inverted hexagonal phases and membrane fusion^36,37^ (Fig. 2C).

To integrate transcriptomic and lipidomic data from *Elovl2*^C234W^ eyecups, Joint Pathway Analysis was performed^38^. Pathway significance was determined by overrepresentation of differentially expressed genes and lipids, and pathway impact scores reflected the topological importance of these changes within each pathway, weighted by relative-betweenness centrality of affected nodes. Among the most significantly enriched pathways were glycerophospholipid metabolism (impact = 0.44, *P* = 3.09E-08), linoleic acid metabolism (impact = 0.19, *P* = 4.45E-04), alpha-linolenic acid metabolism (impact = 0.19, *P* = 9.77E-04), sphingolipid metabolism (impact = 0.3, *P* = 5.98E-03), ether lipid metabolism (impact = 0.15, *P* = 5.62E-03), and arachidonic acid metabolism (impact = 0.15, *P* = 9.73E-03) (Fig. 2D). These integrated results underscore the complex interplay between lipid metabolic dysregulation and transcriptional remodeling in driving natural and ELOVL2-influenced retinal aging and the progressive loss of RPE homeostasis.

### ELOVL2 regulates RPE plasma membrane lipid composition *in vitro*

**Fig 3.**
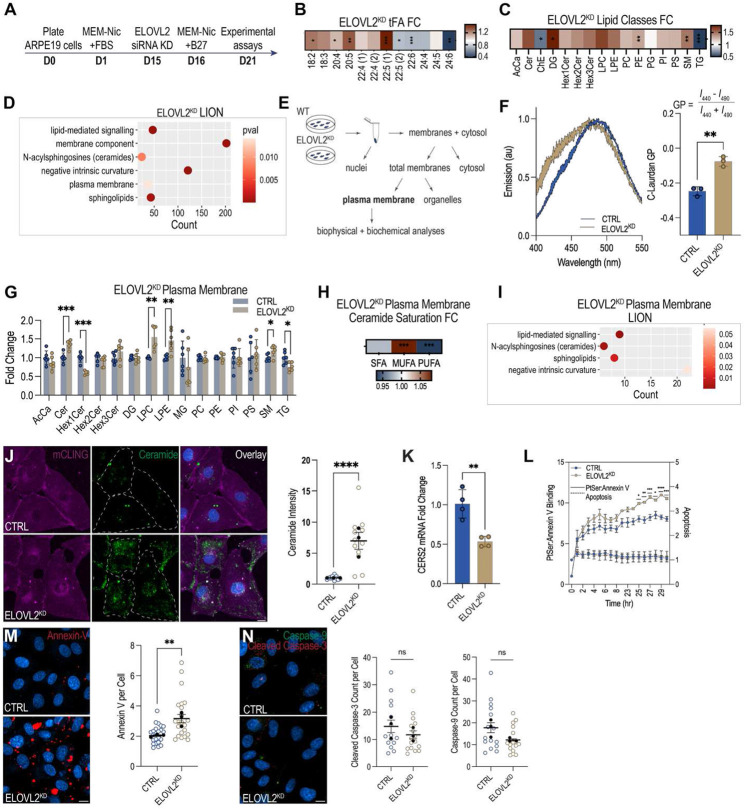
ELOVL2 loss remodels plasma membrane lipidome and induces membrane stress. **a.** Schematic illustration of differentiation of ARPE-19 cells and *ELOVL2* knockdown. **b-c.** Heatmaps showing significantly changed total fatty acids (**b**) and lipid classes (**c**) in whole-cell lipidomics from *ELOVL2*^KD^ cells relative to control (*n* = 4 per group). **d.** LION enrichment analysis of significantly altered lipid species in *ELOVL2*^KD^ cells. **e.** Schematic diagram of the workflow used to isolate plasma membrane fractions for biophysical and lipidomic analyses. **f.** C-Laurdan generalized polarization (GP) analysis of plasma membrane fractions. *Left*: representative spectra for individual samples. *Right*: quantification of GP ratios (*n* = 3 per group). **g.** Significantly altered lipid classes in isolated plasma membrane fractions from *ELOVL2*^KD^ cells relative to control cells (*n* = 6 per group). **h.** Heatmap of ceramide saturation profiles (SFA, MUFA, PUFA) in plasma membrane fractions from control and *ELOVL2*^KD^ cells (*n* = 6 per group). **i.** LION enrichment analysis of significantly altered lipid species in *ELOVL2*^KD^ plasma membrane fractions. **j.** Left: representative confocal images of control and *ELOVL2*^KD^ cells stained with mCLING (membrane marker, pink) and ceramide (green). Scale = 10 μm. Right: quantification of ceramide accumulation in cells. Data are presented as normalized replicate mean ± SEM (closed circles; 3 per group) and fields of view (open circles; 4 per replicate). **k.** qPCR analysis of *CERS2* expression in *ELOVL2*^KD^ cells relative to control (*n* = 4 per group). **l.** Real-time measurement of phosphatidylserine externalization (Annexin V binding, solid lines) and apoptosis activation (dashed lines) (*n* = 3 per group). **m.** Left: representative confocal images of Annexin V (red) in control and *ELOVL2*^KD^ cells. Scale = 10 μm. Right: quantification of Annexin V binding in cells. Data are presented as replicate mean ± SEM (closed circles; 3 per group) and fields of view (open circles; >6 per replicate). **n.** Left: representative confocal images of caspase-9 (green) and cleaved caspase-3 (red) in control and *ELOVL2*^KD^ cells. Scale = 10 μm. Right: quantification of apoptosis activation in cells. Data are presented as replicate mean ± SEM (closed circles; 3 per group) and fields of view (open circles; >4 per replicate). Statistical significance was determined using unpaired t-tests (**b, c, f, g, h, j, k, m, n**) or two-way ANOVA (**l**). *P < 0.05, **P < 0.01, ***P < 0.001, and ****P < 0.0001. Source numerical data are available in Source Data.

To directly investigate the role of ELOVL2 in regulating the lipid composition of RPE membranes independent of photoreceptor-derived input, we cultured ARPE-19 cells differentiated in the presence of nicotinamide^39^. ARPE-19 cells were differentiated in 10 nM nicotinamide, followed by siRNA-mediated knockdown of *ELOVL2* and 6 d in FBS-depleted media supplemented with B27 to provide essential fatty acids, vitamins, antioxidants, and growth factors while minimizing confounding exogenous lipid sources (Fig. 3A). We confirmed differentiation and efficient knockdown of *ELOVL2* by observing a robust 7-fold increase in *RPE65* expression and a 70% decrease in *ELOVL2* expression by qPCR (Fig. S3A). To verify the functional loss of the enzymatic activity of ELOVL2, we quantified total fatty acids and observed an accumulation of its substrate, 22:5, along with a corresponding depletion of LC-PUFAs, including DHA, consistent with impaired elongase activity (Fig. 3B, S3B).

We next performed lipidomic analyses on *ELOVL2*^KD^ cells to assess how the loss of elongase activity impacts the global lipid landscape. This analysis revealed a coordinated deregulation of membrane-enriched lipids, including increased PE and SM, and a redistribution of neutral lipids, marked by elevated diacylglycerol (DG) with reduced TG, alongside a reduction in ChE levels (Fig. 3C, S3C). To contextualize these shifts, we performed LION analysis, which revealed a profile closely resembling the ELOVL2-deficient mouse eyecups. Enriched ontology terms included lipid-mediated signaling, membrane components, and plasma membrane organization (Fig. 3D). Notably, ceramides and sphingolipids were among the most significantly enriched lipid categories, consistent with their roles in regulating membrane curvature, rigidity, and stress responses. These changes indicate a transition in biophysical properties toward a more rigid, curvature-biased membrane state that is highly susceptible to stress^40^.

Because our whole-cell lipidomic results highlighted the enrichment of membrane-associated lipids including ceramides and sphingolipids, we next focused specifically on the purified RPE plasma membrane. Fraction purity was confirmed by Na^+^/K^+^-ATPase enrichment by immunoblot, with no detectable cytosolic (alpha-tubulin) and mitochondrial/organelle (TOM20) markers (Fig. 3E, S3D). Since deviations in membrane fluidity toward excessive rigidity or excessive disorder can compromise cellular function^41^, we first assessed the biophysical properties of the isolated membranes by staining with C-Laurdan, a membrane-phase-sensitive fluorescent dye^42^. C-Laurdan’s fluorescence shifts with lipid packing, emitting at 440 nm in ordered (rigid) membranes and 490 nm in disordered (fluid) membranes. Membrane order was quantified using a Generalized Polarization ratio, where higher GP values represent more ordered membranes and lower GP values indicate more fluid ones. We found that plasma membranes from *ELOVL2*^KD^ cells showed significantly higher GP values, indicating increased membrane order (Fig. 3F).

We next examined how changes in plasma membrane biophysical properties relate to its biochemical makeup by performing global lipidomics analysis on the isolated plasma membrane fractions. PUFA-depleted plasma membrane fractions exhibited decreased levels of Hex1Cer and TGs, increased levels of lysophospholipids, specifically LPC and LPE, and an enrichment in Cer and SM (Fig. 3G). Within the ceramide class, we detected a shift toward MUFA-ceramides and a corresponding decrease in PUFA-ceramides, a pattern similar to the ceramide saturation profile observed in aging eyecups (Fig. 3H, S3E). LION analysis on the significantly changed lipid species revealed a significant enrichment of ceramides, accompanied by an increase in lipids associated with negative intrinsic curvature, which drive inward bending of the membrane and influence vesicle formation, trafficking, and membrane fusion (Fig. 3I).

Consistent with our lipidomics data from isolated plasma membrane fractions, we observed a striking 7-fold accumulation of ceramides in *ELOVL2*^KD^ cells by immunofluorescence (Fig. 3J). Using the membrane marker membrane-binding fluorophore-cysteine-lysine-palmitoyl group (mCLING), ceramide accumulation was visualized at the cell periphery, particularly at the plasma membrane. qPCR analysis also revealed the downregulation of *CERS2*, the enzyme responsible for the synthesis of VLC-ceramides, which likely contributes to the observed shift in the composition of ceramide species (Fig. 3K). These findings indicate a redistribution of ceramides and support the notion that the loss of ELOVL2 activity directly alters plasma membrane composition.

### ELOVL2-deficient RPE cells exhibit plasma membrane stress without triggering apoptosis

Ceramides exert unique biophysical effects on membranes that promote curvature stress and compromise lipid asymmetry^43-45^, and may also cause apoptosis^46,47^. Accordingly, we assessed membrane asymmetry in ELOVL2-deficient cells by measuring externalized phosphatidylserine (PS), an early marker of membrane stress and apoptosis, using two complementary Annexin V binding assays: a real-time assay using RealTime-Glo^™^ and a fluorescent Annexin V conjugate-based assay. As a positive control for both assays, cells were incubated with H2O2 to induce apoptosis^48^. In the real-time assay, *ELOVL2*^KD^ cells exhibited approximately 1.3-fold higher Annexin V binding than control cells at 25 hours, consistent with elevated plasma membrane stress and PS translocation; however, this was not accompanied by a corresponding increase in apoptotic activation, as measured by the lack of DNA dye uptake (Fig. 3L). In contrast, H2O2 treatment triggered Annexin V:PS binding as early as 1 hour, followed by the detection of apoptosis by 23 hours (Fig. S3F). Using the fluorescent Annexin V conjugate, we observed a similar 1.5-fold higher Annexin V binding in *ELOVL2*^KD^ cells compared to controls (Fig. 3M). We further confirmed the lack of apoptosis induction as measured by the absence of cleaved caspase-3 or caspase-9 in control and *ELOVL2*^KD^ cells (Fig. 3N), while H2O2 (6 h) treatment showed robust apoptotic activation (Fig. S3G, H). These results suggest that while *ELOVL2*^KD^ cells experience increased PS externalization and membrane stress, however they do not undergo full apoptotic commitment.

### Lysosome-mediated plasma membrane repair is activated in response to membrane stress

**Fig 4.**
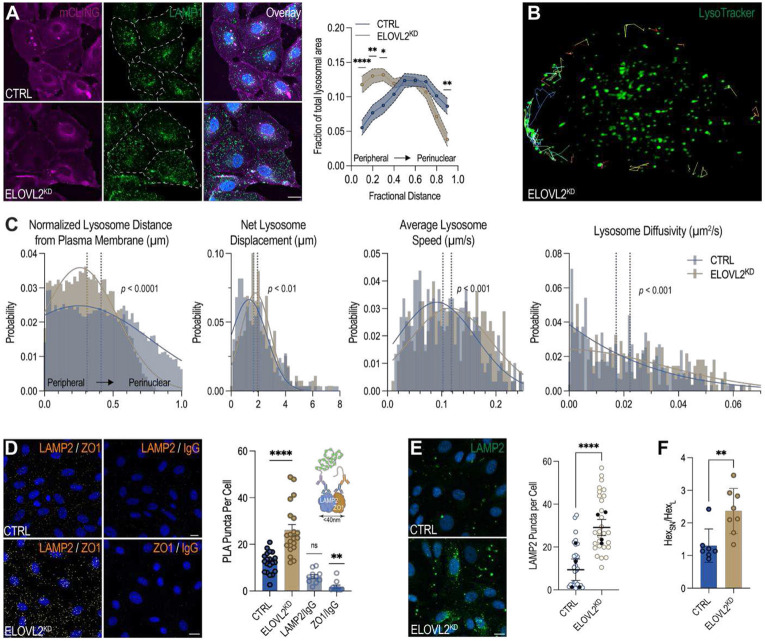
*ELOVL2* deficiency alters lysosomal dynamics and =lysosome-plasma membrane fusion. **a.** Left: representative confocal images of lysosome distribution in control and *ELOVL2*^KD^ cells stained with mCLING (pink) and LAMP1 (green). Scale = 10 μm. Right: radial distribution analysis of lysosomal positioning, where fractional distance = 0 corresponds to the cell periphery and 1 corresponds to the nucleus. Data are presented as mean ± SEM (*n* = 14 fields in control and *n* = 16 fields in *ELOVL2*^KD^, with >4 cells per field, pooled from two biologically independent experiments). **b.** Lattice light-sheet microscopy showing LysoTracker-labeled lysosome tracking in live cells. Only the lysosome tracks within 2 μm of the plasma membrane are shown. **c.** Histogram distribution of lysosome tracks quantifying lysosome distance from the plasma membrane normalized to cell area (left, *n* = 66069 tracks in control and *n* = 51387 tracks in *ELOVL2*^KD^), and peripheral (within 2 μm of the plasma membrane for at least 80% of the observation period) lysosome dynamics (*n* = 298 tracks in control and *n* = 281 tracks in *ELOVL2*^KD^), including displacement (middle left), average lysosome speed (middle right), and diffusivity (right). Distributions were fitted to Gaussian functions, with dashed lines indicating medians. **d.** Left: Representative confocal images of lysosome-plasma membrane interactions by proximity ligation assay (PLA) signals (yellow) using antibodies against LAMP2 and ZO-1. Negative controls include LAMP2/IgG or ZO-1/IgG. Scale = 10 μm. Right: quantification of PLA puncta. Data are presented as mean ± SEM (*n* = 20 fields per group, with >8 cells per field, pooled from two biologically independent experiments). **e.** Left: Representative confocal images of cell surface-exposed LAMP2 (green) in non-permeabilized cells. Scale = 10 μm. Right: quantification of surface LAMP2. Data are presented as replicate mean ± SEM (closed circles; 4 per group) and fields of view (open circles; >6 per replicate). **f.** Lysosomal exocytosis assessed by the β-hexosaminidase release assay, expressed as the ratio of β-hexosaminidase activity in the culture supernatant (SN) to that in the corresponding cell lysate (L). Data are presented as mean ± SEM (*n* = 7 in control and *n* = 8 in *ELOVL2*^KD^). Statistical significance was determined using two-way ANOVA (**a**), two-sided Mann-Whitney U test (**c**), one-way ANOVA with Tukey’s multiple comparison test (**d**), or unpaired t-tests (**e, f**). *P < 0.05, **P < 0.01, ***P < 0.001, and ****P < 0.0001. Source numerical data are available in Source Data.

Membrane repair pathways function to restore plasma membrane integrity that prevent apoptotic progression. To this point, our data show *ELOVL2*^KD^ cells display marked plasma membrane stress and PS externalization, without accompanying cell death. Paradigmatic, lysosomes are recruited to the plasma membrane to reseal sites of damage and restore membrane integrity^49-51^. To explore the role of lysosomes in *ELOVL2*^KD^ cells, we first assessed the spatial distribution of lysosomes by immunostaining for LAMP1 and observed a redistribution of LAMP1-positive lysosomes toward the cell periphery, suggestive of lysosomal recruitment to the plasma membrane^52^ (Fig. 4A, S4A). No significant changes were observed with lysosomal number or diameter (Fig. S4B, C).

To visualize lysosome dynamics, cells were imaged using a light sheet fluorescence microscope, which enables high-speed volumetric acquisition over time with high spatiotemporal resolution^53^ (Fig. 4B). Live cells were labeled with LysoTracker to mark lysosomes and CellMask to delineate the plasma membrane. Lysosome positions were tracked and analyzed relative to cell boundaries, and dynamic parameters were calculated from time-lapse image sequences using particle-tracking software^54^. These analyses confirmed that *ELOVL2*^KD^ cells exhibited an increased population of lysosomes near the plasma membrane indicating that, on average, lysosomes were concentrated within the peripheral ~30% of the cell, consistent with potential recruitment toward sites of membrane repair (Fig. 4C). Furthermore, peripheral lysosomes at the plasma membrane were significantly more dynamic in *ELOVL2*^KD^ cells relative to control cells, as indicated by increased net displacement (1.650 ± 0.082 μm in control, and 1.939 ± 0.082 μm in *ELOVL2*^KD^, *P =* 0.003), average speed (0.102 ± 0.003 μm/s in control, and 0.118 ± 0.003 μm/s in *ELOVL2*^KD^, *P =* 0.0004) and diffusivity (0.017 ± 0.001 μm^2^/s in control, and 0.022 ± 0.001 μm^2^/s in *ELOVL2*^KD^, *P =* 0.0005). These data align with an enhanced lysosomal mobilization toward the plasma membrane for repair (Fig. 4C).

To directly test whether lysosomes were engaging in membrane fusion events, we performed a proximity ligation assay (PLA) using antibodies against LAMP2, a lysosomal membrane protein, and ZO-1, a tight junction-associated marker of the plasma membrane (Fig. 4D). This approach detects protein-protein interactions in proximity (<40 nm), indicative of lysosomal docking at the plasma membrane. *ELOVL2*^KD^ cells displayed a significant increase in PLA puncta, reflecting enhanced lysosome–plasma membrane contact (Fig. 4D). No signal was detected in negative controls using either LAMP2 and immunoglobulin G (IgG), or ZO-1 and IgG (Fig. 4D). Importantly, immunofluorescence staining revealed no redistribution or intracellular translocation of ZO-1, confirming that the PLA signal does not arise from ZO-1 relocalization into the cell but rather from lysosomes approaching and engaging the plasma membrane (Fig. S4D).

Lysosome-mediated membrane patching leads to the transient appearance of lysosomal membrane proteins at the plasma membrane surface^55^. To assess this phenomenon, immunofluorescence microscopy was performed on non-permeabilized cells using an antibody against the luminal epitope of LAMP2, which demarcates the exposure of lysosomes at the plasma membrane. Consistent with enhanced lysosomal fusion, *ELOVL2*^KD^ cells exhibited increased LAMP2 at the cell surface compared to controls (Fig. 4E). As an orthogonal method, surface exposed LAMP2 was evaluated by flow cytometry and similarly showed that *ELOVL2*^KD^ cells displayed a higher fraction of LAMP2^+^ cells (Fig. S4E).

Lysosome-mediated plasma membrane repair involves the fusion of lysosomes with the cell surface, which releases lysosomal contents to the extracellular space^56^. To test this, we evaluated β-hexosaminidase, a lysosomal hydrolase, in the culture supernatant with 4-Methylumbelliferyl N-acetyl-β-D-glucosaminide, a fluorogenic substrate that releases a fluorescent product upon β-hexosaminidase-mediated enzymatic cleavage. β-hexosaminidase enzymatic activity was quantified within each fraction and revealed a significantly elevated supernatant-to-lysate activity ratio in *ELOVL2*^KD^ cells, indicating an extracellular release of lysosomal enzymes (Fig. 4F, S4F). These data suggest that ELOVL2 depletion leads to lysosomal exocytosis in response to plasma membrane stress.

### Lysosomal engagement in membrane repair limits phagocytic capacity upon ELOVL2 loss

Homeostatic lysosomal function is essential for the degradation of phagocytosed photoreceptor outer segments (POS)^57,58^. Whether the continual recruitment of lysosomes to the plasma membrane for repair impacts homeostatic lysosomal function is unknown. To assess whether these functions were compromised *in vivo*, we first quantified POS phagocytosis in 18-month-old wild-type and *Elovl2*^*C234W*^ mutant mice. RPE flatmounts were collected at time points capturing the peak and progression of the daily phagocytic burst in mice^59,60^ and stained with the rod outer segment marker 1D4 (Fig. S4G). Wild-type animals displayed the expected temporal dynamics of phagocytosis where abundant internalized 1D4-positive phagosomes are present at the earliest time point (ZT1), while signal declines as phagosomes are degraded at later times (ZT4/ZT8) (Fig. S4G). In contrast, *Elovl2*^*C234W*^ mutants exhibited significantly fewer internalized phagosomes (ZT1), indicative of impaired phagocytic uptake (Fig. S4G). Furthermore, phagosome size was significantly larger in mutants, consistent with delayed processing or stalled lysosomal degradation (Fig. S4G). These data indicate that disrupting Elovl2 activity leads to multiple defects *in vivo*.

To further evaluate phagocytosis, we performed a flow cytometry–based uptake assay^61^. Cultured ARPE-19 cells were incubated with fluorescently labeled-POS (FITC-POS) and phagocytic uptake was quantified. Consistent with the *in vivo* defects observed in the RPE of *Elovl2*^*C234W*^ mutant mice, *ELOVL2*^KD^ cells exhibited a significant reduction in the percentage of FITC-positive cells compared with control cells, indicating impaired phagocytosis (Fig. S4H). These *in vivo* and *in vitro* data demonstrate that loss of ELOVL2 compromises phagocytic function in RPE cells, which aligns with a sustained lysosomal recruitment to the plasma membrane.

### ELOVL2 deficiency triggers membrane stress and polarized lysosomal responses in iPSC-RPE

**Fig 5.**
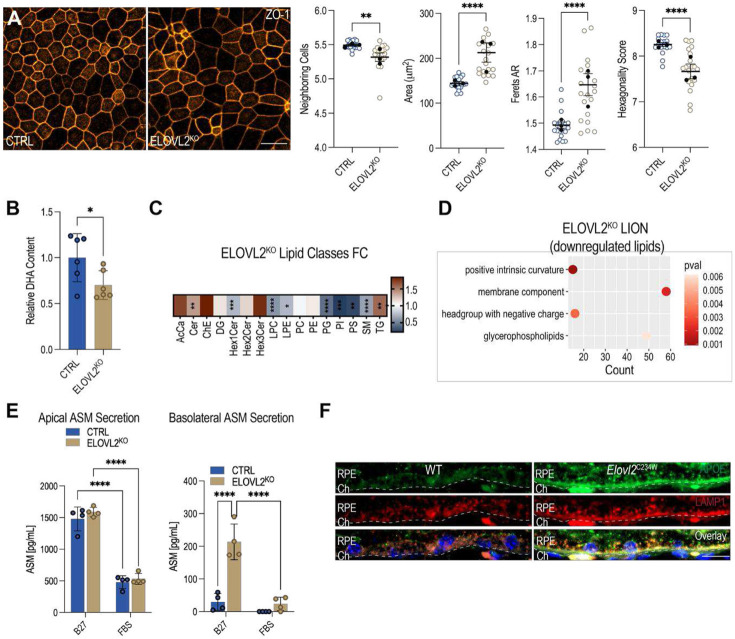
ELOVL2 depletion triggers basolateral lysosome-plasma membrane remodeling. **a.** Left: representative confocal images of ZO-1-labeled tight junctions (orange) in control and *ELOVL2*^KO^ iPSC-RPE monolayers. Scale = 20 μm. Right: quantitative morphometric analysis performed using ReSHAPE, including measurements of neighboring cells, cell area, Feret’s aspect ratio, and hexagonality score. Data are presented as replicate mean ± SEM (closed circles; 3 per group) and fields of view (open circles; >6 per replicate). **b.** Fold change in DHA abundance in *ELOVL2*^KO^ cells relative to control iPSC-RPE, normalized to total lipid content and internal standards. Data are presented as mean ± SEM (*n* = 6 per group). **c.** Heatmap of differentially abundant lipid classes in *ELOVL2*^KO^ iPSC-RPE (*n* = 6 per group). **d.** LION enrichment analysis of significantly downregulated lipids (FC > −1.5, *P* < 0.05) in *ELOVL2*^KO^ iPSC-RPE. **e.** Acid sphingomyelinase (ASM) protein levels measured by ELISA in conditioned media collected from the apical (left) and basolateral (right) compartments of polarized iPSC-RPE monolayers cultured in FBS- or B27-containing media. Data are presented as mean ± SEM (*n* = 4 per group). **f.** Representative retinal cross-sections from 18-month-old wild-type and *Elovl2*^C234W^ mice stained for APOE (green) and LAMP1 (red). Scale = 10 μm. Statistical significance was determined using unpaired t-tests (**a, b, c**) or two-way ANOVA (**e**). *P < 0.05, **P < 0.01, ***P < 0.001, and ****P < 0.0001. Source numerical data are available in Source Data.

Induced pluripotent stem cell (iPSC)-derived RPE are widely regarded as a faithful model of human RPE, capable of recapitulating many structural, functional, and physiological properties of native tissue^62,63^. Building on our findings in ARPE-19 cells and mouse RPE, we generated *ELOVL2*-knockout (*ELOVL2*^KO^)-iPSCs via CRISPR-Cas9^64^, and differentiated wild-type and *ELOVL2*^KO^ iPSCs into RPE using the STAR protocol^65^. We first performed morphometric analysis using the REShAPE pipeline^26^. High-resolution imaging of confluent ZO-1-stained monolayers revealed significant disruptions in cell morphology in *ELOVL2*^KO^ iPSC-RPE, as quantified by a decrease in the number of neighboring cells, an increase in cell area and Feret’s ratio, and an overall decrease in hexagonality score (Fig. 5A). Consistent with loss of ELOVL2 enzymatic activity, fatty acid analysis demonstrated a significant reduction in DHA content in *ELOVL2*^KO^ iPSC-RPE compared with wild-type cells (Fig. 5B). Lipidomic profiling of *ELOVL2*^KO^ iPSC-RPE revealed a significant accumulation of TGs, in addition to increased levels of Cer and the depletion of SM, suggesting enhanced SM-to-Cer conversion through acid sphingomyelinase (ASM) activity (Fig. 5C, S5A). Conversely, levels of Hex1Cer and many phospholipid classes including LPC, LPE, phosphatidylglycerol (PG), phosphatidylinositol (PI) and PS, were decreased, indicating compromised membrane homeostasis and altered phospholipid remodeling (Fig. 5C, S5A). LION analysis of significantly downregulated lipids identified a strong overrepresentation of membrane-associated components (Fig. 5D). These changes mirror the cytoskeletal and epithelial integrity defects observed in vivo and underscore the importance of ELOVL2 in RPE health.

Lysosomal exocytosis is enabled by the activity of ASM, a lysosomal enzyme that is externalized and hydrolyzes sphingomyelin into ceramide, generating membrane domains that facilitate repair and remodeling^66^. Therefore, we examined whether this process contributes to plasma membrane remodeling in polarized iPSC-RPE cells. ASM protein levels were quantified within the apical and basolateral compartments using ELISA. First, we found that ASM release was higher in the apical than the basolateral compartment under both media conditions (Fig. 5E). Second, overall ASM secretion was greater in both CTRL and *ELOVL2*^KO^ cells in DHA-depleted medium compared to FBS medium (Fig. 5E). Third, *ELOVL2*^KO^ cells exhibited a selective increase in basolateral ASM release in B27 medium, which was significantly decreased when cells were cultured in FBS medium (Fig. 5E). Given that lysosomal exocytosis diverts lysosomes from their degradative roles, we next asked whether enhanced membrane repair activity was accompanied by impaired autophagic capacity in iPSC-RPE. *ELOVL2*^KO^ iPSC-RPE exhibited a decreased LC3-II to LC3-I ratio, indicative of reduced autophagosome formation and impaired autophagy^67^ (Fig. S4I). Furthermore, consistent with reduced autophagy^68-70^, *mTOR* transcript levels were also increased in *ELOVL2*^KD^ cells (10-fold) relative to control cells (Fig. S4J). These findings indicate that membrane stress-induced lysosomal exocytosis in ELOVL2-deficient RPE is coupled to polarized ASM secretion, favoring the basolateral surface and potentially impacting ceramide accumulation and membrane remodeling at the RPE-Bruch’s membrane-choroid interface.

### *In vivo* basal lysosomal trafficking and lipoprotein accumulation in ELOVL2 deficient RPE

Consistent with this polarized increase in ASM secretion, we observed parallel evidence of enhanced lysosome-plasma membrane engagement at the basal surface *in vivo*. In retinal cross-sections from 18-month-old *Elovl2*^*C234W*^ mutant mice, LAMP1 immunoreactivity was markedly redistributed toward the basal aspect of the RPE, in contrast to age-matched wild-type controls, where LAMP1 was more evenly distributed throughout the cytoplasm (Fig. 5F). Notably, *Elovl2*^*C234W*^ mutants also exhibited increased APOE accumulation at the basal RPE surface, a feature associated with aging and sub-RPE deposit formation^71,72^.

The basal redistribution of lysosomal membrane proteins mirrors the basolateral ASM release detected in iPSC-RPE and indicates that ELOVL2 deficiency promotes directional lysosomal trafficking and exocytic activity toward the RPE-Bruch’s membrane interface. Together, these findings support a model in which chronic membrane stress in ELOVL2-deficient RPE drives polarized lysosomal exocytosis, localized membrane remodeling, and the basal accumulation of lipoprotein-rich material—processes that may contribute to pathological alterations in the outer retina and features reminiscent of early AMD.

### Plasma membrane stress and lysosome trafficking are directly regulated by ELOVL2 enzymatic activity

**Fig. 6.**
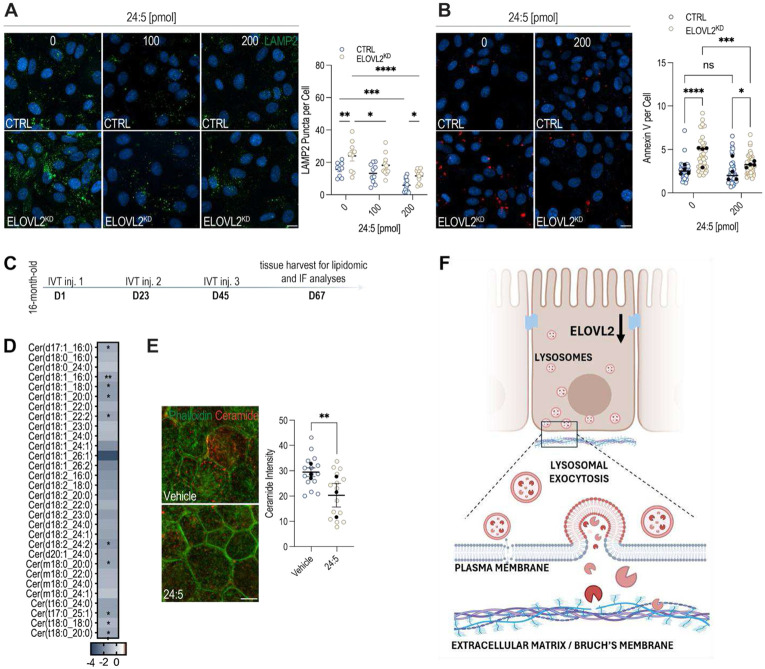
Supplementation with ELOVL2-derived product rescues age-related phenotypes. **a.** Left: representative confocal images of non-permeabilized control and *ELOVL2*^KD^ cells treated with increasing amounts of 24:5n-3 (0, 100, 200 pmol) for 4 d and stained for cell surface-exposed LAMP2 (green). Scale = 10 μm. Right: quantification of surface-LAMP2 after 24:5n-3 supplementation. Data are presented as mean ± SEM (*n* > 8 fields per condition), pooled from two biologically independent experiments. **b.** Left: representative confocal images of Annexin V staining (red) in control and *ELOVL2*^KD^ cells treated with 100 pmol 24:5n-3 for 4 d. Scale = 10 μm. Right: quantification of Annexin V after 24:5n-3 supplementation. Data are presented as replicate mean ± SEM (closed circles; 4 per group) and fields of view (open circles; >9 per replicate). **c.** Schematic diagram of monthly intravitreal 24:5n-3 supplementation in 16-month-old mice for 3 months. **d.** Heatmap of ceramide lipid species in eyecups from 24:5n-3-supplemented mice relative to eyecups from vehicle-injected mice (*n* = 3 per group). **e.** Left: representative confocal images of RPE flatmounts from 24:5n-3-supplemented eyes, stained for ZO-1 (green) and ceramide (red). Scale = 10 μm. Right: quantification of ceramide fluorescence in RPE flatmounts from 24:5n-3-supplemented eyes. Data are presented as replicate mean ± SEM (closed circles; 3 per group) and fields of view (open circles; 5 per replicate). **f.** Proposed mechanism of changes occurring in aged RPE. Statistical significance was determined using two-way ANOVA (**a, b**) or unpaired t-tests (**d, e**). *P < 0.05, **P < 0.01, ***P < 0.001, and ****P < 0.0001. Source numerical data are available in Source Data.

To determine whether the observed phenotypes resulted directly from the loss of ELOVL2 enzymatic activity, we tested whether supplementing the ELOVL2 product could mitigate membrane stress and lysosome-mediated membrane remodeling. Differentiated ARPE-19 cells were treated daily with 24:5n-3 for 4 d. Cells were then assayed for externalized LAMP2 on non-permeabilized cells and for Annexin V binding in live cells. Both control and *ELOVL2*^KD^ cells exhibited a dose-dependent reduction of surface LAMP2 exposure with 24:5n-3 supplementation (Fig. 6A). The decrease in surface LAMP2 was statistically significant in *ELOVL2*^KD^ cells at both 100 and 200 pmol compared to vehicle and in control cells at 200 pmol (Fig. 6A). For Annexin V binding, a significant reduction was observed in *ELOVL2*^KD^ cells treated with 200 pmol 24:5n-3, whereas control cells showed no difference (Fig. 6B). These results indicate that plasma membrane stress and lysosomal exocytosis are directly dependent on optimal ELOVL2 activity.

In our previous work, we showed that intravitreal injection of 24:5n-3 improves vision in aged animals and restores lipid composition in the retina^11^. To further identify the lipidomic changes in the RPE upon such supplementation, 16-month-old wild-type mice were treated with 24:5n-3 through monthly intravitreal injections (three doses total) (Fig. 6C). Lipidomic analysis of vehicle-versus 24:5n-3-treated eyecups revealed a consistent reduction in multiple ceramide species in the supplemented eyecups (Fig. 6D, S5B). In addition, ceramide immunofluorescence of RPE flatmounts showed significantly lower ceramide signal intensity in 24:5n-3-supplemented eyes, indicating decreased accumulation of membrane-stress-associated sphingolipids (Fig. 6E). Together, these lipidomic and imaging data demonstrate that lipid imbalance in the RPE plasma membrane during aging is directly dependent on the availability of ELOVL2’s PUFA-elongation products, underlying the central role of balanced ELOVL2 expression in healthy aging.

## DISCUSSION

Here, we identify a central role for ELOVL2-mediated PUFA elongation in maintaining plasma membrane composition, biophysical properties, and structural integrity in the RPE. Loss of ELOVL2 activity disrupts lipid balance, driving ceramide accumulation and leading to the recruitment of lysosomes to the plasma membrane, where they engage in a sustained repair program to counteract chronic membrane stress (Fig. 6F).

Our analysis of human RPE at an advanced age revealed a consistent enrichment of genes associated with plasma membrane and endomembrane system components, highlighting that membrane-associated processes are particularly susceptible to age-dependent transcriptional remodeling. Complementary analyses of eyecups from aged mice confirmed that these signatures are conserved across species, establishing membrane remodeling as a hallmark of RPE aging.

Loss of PUFA elongation in eyecups from *Elovl2*^*C234W*^ mutant mice led to a marked accumulation of ceramides and sphingomyelins, accompanied by the depletion of LC- and VLC-PUFAs (Fig. 2C, D). These compositional shifts have profound implications for membrane biophysical properties. The increased GP values observed in plasma membrane fractions from ELOVL2-deficient cells are consistent with a more ordered membrane environment, most likely due to elevated ceramide levels and reduced long-chain PUFA incorporation in the plasma membrane (Fig. 3F). High membrane fluidity and the capacity of lipids to adopt nonlamellar topologies such as the inverted hexagonal (HII) phase are thought to be essential for membrane fusion and vesicular trafficking^73^; thus, loss of these properties likely compromises dynamic membrane remodeling in the RPE, affecting processes such as phagocytosis and autophagy, vesicle trafficking, and intercellular barrier maintenance.

Notably, despite exhibiting increased plasma membrane stress and the externalization of PS, ELOVL2-deficient RPE cells did not undergo apoptotic commitment. Instead, we observed a redistribution of lysosomes to the cell periphery, increased surface LAMP2 exposure, and elevated β-hexosaminidase and ASM release (Fig. 4A, C, D, E, S4E, 5E). These findings reveal a compensatory mechanism of plasma membrane repair that maintains cell viability despite persistent lipid imbalance. Specifically, damaged membranes are resealed via lysosome-plasma membrane fusion. Our data show that ELOVL2 deficiency, by altering membrane lipid composition and promoting ceramide accumulation, creates a state of chronic membrane stress that continually engages this repair pathway. This adaptive response likely enables the RPE to preserve structural integrity under metabolic stress but may also contribute to extracellular matrix remodeling and sub-RPE deposits characteristic of aging and AMD. Specifically, the increase of ASM secretion as a component of this lysosomal response provides a mechanistic link between intracellular lipid stress and extracellular space remodeling. ASM, when externalized during lysosomal exocytosis, hydrolyzes SM to ceramide at the cell surface, generating membrane domains with negative curvature, as previously observed in diseased RPE^74-79^, that can further promote membrane rupture^66,80^. Elevated basolateral release of ASM in ELOVL2-deficient and aged RPE thus represents a coordinated remodeling response that may have dual outcomes: facilitating membrane repair while contributing to sub-RPE ceramide accumulation.

The polarized nature of this secretion further suggests that membrane stress responses in the RPE are spatially regulated, potentially influencing interactions with Bruch’s membrane and the choroid and contributing to drusen formation/deposition. Specifically, the chronic release of lysosomal contents—including undigested material and enzymes involved in protein and lipid degradation—may promote remodeling of the extracellular environment and accumulation of sub-RPE debris. Consistent with this interpretation, our data show colocalization of APOE with LAMP1 in the RPE of *Elovl2*^*C234W*^ mutant mice, coinciding with elevated levels of sub-RPE APOE-containing deposits (Fig. 5F).

Our data further indicates that under restricted nutrient conditions (B27- containing media), wild-type cells also exhibit plasma membrane stress, as evidenced by detectable LAMP2 externalization and Annexin V binding (Fig. 3M, 4E). Moreover, when cultured in defined minimal medium, wild-type iPSC-RPE, similar to ELOVL2-deficient cells, show increased lysosomal exocytosis at the apical surface (Fig. 5E). These findings suggest that low-nutrient conditions, such as those arising from sub-RPE deposits that impede exchange with the choroid, may exacerbate membrane stress and promote lysosome-mediated remodeling. Over time, this process could contribute to the formation of subretinal debris, potentially disturbing the interaction between the RPE and photoreceptors.

Lysosomes are increasingly recognized not only as degradative organelles, but also as central signaling hubs that coordinate nutrient sensing, stress responses, and longevity pathways across multiple tissues^81-86^. Age-related loss of lysosomal activity, often assessed by reduced autophagy or phagocytosis, has been reported in numerous studies^87-91^. Mechanistically, aging has been associated with an increase in the pH of lysosomes, which can impair enzymatic function^92^. Here, we propose an additional mechanism: lysosomes that remain functional are hijacked for compensatory plasma membrane repair, helping cells maintain integrity and avoid death. Importantly, our findings do not exclude contributions from other age-related lysosomal perturbances.

While the role of ELOVL2 in the RPE has not been previously studied, the importance of VLC-PUFAs for RPE health and function has been documented in several studies, including Stargardt disease models^93-95^. Although the RPE primarily recycles rather than synthesizes PUFAs longer than 22 carbons^96^, endogenous production of DHA appears critical for maintaining effective recycling. Furthermore, age-related accumulation of debris at the RPE-Bruch’s membrane interface may limit nutrient access from the choroid, increasing reliance on endogenously produced PUFAs such as DHA to support RPE function. Consistent with our hypothesis, supplementation with 24:5n-3, the direct product of ELOVL2, improved vision^11^, reduced membrane stress and attenuated lysosome-mediated remodeling, placing ELOVL2 at the center of retinal health maintenance.

Taken together, our results support a model in which the age-dependent decline in PUFA elongation disrupts the balance between membrane flexibility and stability, initiating a compensatory cycle of membrane stress and repair. This cycle is characterized by ceramide accumulation, lysosomal recruitment to the plasma membrane, and ASM-mediated membrane remodeling. While this response mediates short-term cell survival, chronic activation of this repair mechanism may drive long-term structural changes, the formation of extracellular deposits, and functional decline. Beyond the retina, *ELOVL2* encodes a key enzyme in the elongation of LC- and VLC-PUFAs, including DHA, which are essential for neural, retinal, and cardiovascular health. Its prominent hepatic expression suggests an additional role in supplying PUFAs to peripheral tissues, raising the possibility that similar age-related mechanisms operate in other post-mitotic epithelia that rely on dynamic membrane maintenance.

## METHODS

### Cell Culture

#### ARPE-19

ARPE-19 (ATCC CRL-2302) cells were maintained in DMEM/F12 + GlutaMAX supplemented with 10% FBS at 37°C in a humidified incubator with 5% CO_2_. For differentiation, ARPE-19 cells were cultured in MEM-Nic medium (MEM alpha with GlutaMAX, 1% FBS, 1% Penicillin/Streptomycin, 1% N1 supplement, taurine (0.25 mg/mL), hydrocortisone (20 ng/mL), triiodothyronine (0.013 ng/mL), and 10 mM nicotinamide) with media changes three times per week for two weeks following a previously published protocol^39^.

To reduce the source of exogenous lipids, cells were cultured in a defined minimal medium consisting of the same MEM-Nic media used for standard maintenance with chemically defined, serum-free supplement B27 (Gibco). Cells were maintained in B27 medium for 6 d prior to downstream assays.

#### iPSC-RPE

##### Cell culture media and composition

###### N2 supplement (1x).

100μg/mL Optiferrin (Invitria, #777TRF029), 5μg/mL insulin (Sigma, #4512), 6.3ng/mL progesterone (Sigma, #P8783), 16.11μg/mL putrescine (Sigma, #P5780), 5.2 ng/mL sodium selenite (Sigma, #S9133) in DMEM/F12 (Gibco #11330-057) as previously described^97^.

###### B8 stem cell medium.

DMEM/F12 with HEPES (Corning #10-092-CV), 200μg/ml L-Ascorbic acid 2-phosphate trisodium salt (Fujifilm Wako, #011-28471), 5μg/ml insulin (Sigma #4512), 5μg/ml Optiferrin (Invitria #777TRF029), 20ng/ml sodium selenite (Sigma #S9133), 40ng/ml fibroblast growth factor 2-G3 (FGF2-G3) (Qkine #Qk053), 0.1 ng/ml neuregulin 1 (NRG1; Peprotech #100-03), 0.1ng/ml transforming growth factor beta-1 (TGFβ1; Qkine, #Qk010) as previously described^98^.

###### B27 Supplement for IPSCs (1x).

B27 medium was prepared according to its original formulation, with minor modifications^99^. 2.5mg/mL bovine serum albumin - BSA (FA and IgG-free, Fraction V; Sigma #A4919), 2.5μg/mL catalase (Sigma #C40), 2.5μg/mL superoxide dismutase (Sigma #S5395), 3.125 μg/mL human recombinant insulin (Sigma #4512), 5μg/mL Optiferrin (Invitria #777TRF029), 1.0μg/mL reduced glutathione (Sigma# G6013), 16.1μg/mL putrescine·HCl (Sigma #P5780), 14.35ng/mL sodium selenite (Sigma# S5261), 2ng/mL triiodothyronine (Sigma #T6397), 6.3ng/mL progesterone (Sigma #P8783), 20ng/mL corticosterone (Sigma #C2505), 2.0μg/mL L-carnitine (Sigma #C0283), 1.0μg/mL ethanolamine (Sigma #E9508), 15μg/mL D-galactose (Sigma #G0625), 1.0μg/mL linoleic Acid (Sigma #L1012), 1.0μg/mL linolenic Acid (Sigma #L2376), 47ng/mL lipoic acid (Sigma #T1395), 100ng/mL retinyl acetate (Sigma #R7882), 1μg/mL ɑ-tocopherol (Sigma #T3251), 1.0μg/mL ɑ-tocopherol Acetate (Sigma #T3001), 10ng/mL biotin (Sigma #B4639), 1.0μg/mL pipecolic acid (Sigma #P2519).

###### RIM (Retinal Induction Medium).

DMEM/F12 (Gibco #11330), 200 μM ascorbic acid (Sigma#A4544), 1x B27 Supplement, 1.5% Knockout Serum Replacement (KSR; Gibco #10828010), 1x non-essential amino acids (NEAA; Gibco #11140050), 1x N2 supplement, 1x sodium pyruvate (Gibco #11360-070), 10 ng/mL AF-IGF-1 (Qkine #Qk047), 5μM CKI-7 dihydrochloride (Sigma #1177141-67-1), 100 nM LDN-193189 (Med Chem Express #HY-12071A), 10μM SB431542 (Ambeed #A172016).

###### RDM (Retinal Differentiation Medium).

DMEM/F12 (Gibco #11330-057), 200 μM ascorbic acid, 1x B27 Supplement, 1.5% KSR, 1x NEAA, N2 supplement, 1x sodium pyruvate, 10 ng/mL AF-IGF-1 (Qkine #Qk047), 5μM CKI-7 dihydrochloride (Sigma#1177141-67-1), 100 nM LDN-193189 (Med Chem Express #HY-12071A), 1μM PD0325901 (Med Chem Express #HY-10254), 10μM SB431542 (Ambeed #A172016).

###### RMNA (Retinal Media with Nicotinamide and Activin-A).

DMEM/F12 (Gibco #11330-057), 200 μM ascorbic acid (Sigma #A4544), 1x B27 Supplement, 1.5% KSR, 1x NEAA, 1x N2 supplement, 1x sodium pyruvate, 100ng/mL Activin-A (Qkine #Qk001), 10mM nicotinamide (Sigma #N0636).

###### RPE-MM (RPE maturation media).

MEM alpha (Gibco #12571-063), 1x non-essential amino acids (NEAA; Gibco #11140050), 1x N2 supplement, 1x sodium pyruvate, 5% value grade heat-inactivated Fetal Bovine Serum (Gibco #A5256701), 13ng/mL T3 (Sigma #T5516), 250μg/mL taurine (Sigma #T8691).

#### Stem cell maintenance

EP1.1 iPSCs (female)^100^ were used for the following study with approval from the UC San Diego and UC Irvine Institutional Review Boards. Stem cells were maintained antibiotic-free on 1% (vol/vol) Matrigel (MG)-GFR^™^ (Corning #354230) coated dishes at 37 °C under normoxia (5% CO2/20%O2) in mTSR1 or B8 media^98^ as previously described. Cells were clump passaged every 4–6 d, with 0.5mM EDTA in PBS for 7 min. After 24-48 h, cells were fed with mTSR1 or B8 alone.

##### Creation of knockout iPSC lines.

ELOVL2 knockout was achieved EP1.1 iPSCs as previously described^101,102^. Briefly, iPSCs were dissociated using Accutase (Sigma #A6964) for 12 minutes at 37°C. After a brief centrifugation, the media was removed, and the cells were incubated on ice for 15 minutes. Immediately before electroporation, cells were resuspended in homemade R-buffer (Sucrose-based buffer: 250 mM sucrose and 1 mM MgCl_2_ in Ca^++^/Mg^++^ free DPBS (Gibco # 14190)) containing RNPs targeting ELOVL2. Electroporation was performed at 1,300 V; interval, 20 ms; 1 pulse using the Neon Transfection system (Invitrogen). Cells were immediately transferred to 1% Matrigel-coated plates in B8 supplemented with 2μM thiazovivin (Cayman Chemical #14245) to improve cell survival. For each genomic modification, RNPs for gene-editing were comprised of 4 μg of Cas9 protein (IDT) and ~0.4 μg of RNA targeting the *ELOVL2* gene (IDT). Electroporated iPSCs were single-cell passaged for clonal isolation, followed by genomic DNA extraction with Quick Extract (Biosearch Tech-formerly Lucigen), PCR amplification of the target region with Phusion Polymerase (Thermo # F548L), and oligos flanking the insert site (Forward: TTC CTA CTC AAA CCT TGA ACA GAT GCC AGA and Reverse: ACT TGG TAG AGG CAT CTG CTA CGT GGA TGT). Amplicons were Sanger sequenced with the nested primer (GCC CTC TAT CTG GAA GGA GAA) and aligned using Geneious Prime (Biomatters).

###### Generation of RPE from iPSC

To generate RPE from iPSC, cells were dissociated with TrypLE^65^ for 10 min, quenched in B8T, and centrifuged for 5 min at 80g as previously described^65^. The cell pellet was resuspended in 500μL of B8T, counted by a hemocytometer, and plated into a Matrigel-coated 12-well TC-treated plate at a density of 110,000 cells per well in B8 plus 2 μM thiazovivin. The following day, B8T was aspirated and replaced with B8 media. Day 0 begins 48 h after the initial seeding of cells. From D0 to D2, cells were fed each day with Retinal Induction Media (RIM). From days 2 through 10, cells were fed Retinal Differentiation Media (RDM) daily. From day 11 to day 20, cells were fed with Retinal Media with Nicotinamide and Activin-A (RMNA). From D21 onwards, cells were fed with RPE Maturation Media (RPE-MM) until pigmented clusters emerged, typically around day 30. To enrich pigmented cell count, non-pigmented cells were scraped away with a flame-sterilized glass rod. Regions enriched for pigmented cells were carefully lifted off the plate using a flame-sterilized tungsten needle, gently aspirated using a P200 pipette, and transferred to a fresh TC-treated 1% Matrigel-coated 12-well plate in RPE-MM plus 2 μM thiazovivin. The following day, media was replaced with RPE-MM without thiazovivin and fed every other day from then on. These colonies were allowed to expand for 2-3 weeks, and then passaged with TrypLE into 12-well plates at a density of 100,000 cells per well. RPE was passaged each month (but no longer than two months as needed), up to passage 5, until enough RPE cells were present for plating on the required number of 12-well, 0.4 μm pore-size Transwell plates (Corning #3460) used for RPE maturation. For Transwells, cells were passaged with TrypLE as described above and plated at a density of 200,000 cells per well in the Matrigel-coated Transwell insert in RPE-MM plus thiazovivin. Media was replaced the following day with RPE media lacking thiazovivin and fed every other day for a total of 9 months from the first passage to the time of analysis in Transwell plates.

### Electroporation

ARPE-19 cells and differentiated ARPE-19 cells were transfected using the Neon Transfection System (Thermo Fisher Scientific) according to the manufacturer’s instructions. Briefly, cells were resuspended in Neon Resuspension Buffer R and electroporated with either a scrambled control siRNA or an *ELOVL2*-targeting siRNA (10 nM). Electroporation was performed using the Neon 10 μL or 100 μL tip with the following optimized parameters for ARPE-19 cells: 1350 V, 20 ms pulse width, 2 pulses. Immediately after electroporation, cells were transferred into pre-warmed culture medium and allowed to recover under standard culture conditions (37°C, 5% CO_2_). The next day, the medium was replaced with serum-depleted medium containing B27 supplement.

### Bulk RNA sequencing

#### Sample collection and preparation

Fresh mouse eyecups were dissected from each mouse eye. ARPE-19 cells were harvested by trypsinization and pelleted. For both sample types, total RNA was extracted using the RNeasy Plus Mini Kit (Qiagen) following the manufacturer’s instructions. RNA quantity and quality were assessed using the Qubit RNA HS Assay kit (Thermo Fisher Scientific) and the Agilent Bioanalyzer RNA 6000 Pico kit (Agilent Technologies), respectively.

#### Library construction and sequencing

RNA sequencing libraries were prepared using the Illumina TruSeq RNA Library Prep kit. Paired-end sequencing was performed on the NovaSeq 6000 System using the Flow Cell Type S4, generating paired-end reads with a length of 100 base pairs and approximately 30 million reads per sample.

#### Bioinformatics analysis

Raw sequencing reads were preprocessed to remove adapter sequences and low-quality bases using Trimmomatic. Cleaned reads were mapped to the appropriate reference genome (mm10 for mouse eyecups, GRCh38 for ARPE-19 cells) using HISAT2. Gene-level expression counts were quantified using featureCounts. Differential expression analysis was performed using DESeq2, and genes with an adjusted p-value < 0.05 and ∣fold change∣ > 1.5 were considered differentially expressed.

### Gene set enrichment analysis (GSEA)

Gene Set Enrichment Analysis (GSEA) was conducted using the GSEA software (v4.2.3)^103^. Differentially expressed genes identified by DESeq2 were ranked using the signal-to-noise ratio rank metric. Enrichment analysis was performed using predefined gene sets from databases such as Gene Ontology (GO), the Molecular Signatures Database hallmark gene set, Reactome, and Wikipathways^104-107^. Statistical significance of enrichment was determined using a weighted (*p*=1) scoring calculation scheme with 1000 permutations. Gene sets with sizes larger than 500 and smaller than 5 were excluded from the analysis.

### Joint pathway analysis of lipidomics and transcriptomics

To identify pathways jointly altered at the transcriptional and lipidomic levels, we performed integrated pathway analysis using MetaboAnalyst 6.0^38^. Differentially expressed genes from bulk RNA sequencing *(P < 0.05)* and significantly altered lipid species from global lipidomics (*P < 0.05*) were uploaded to the MetaboAnalyst joint pathway analysis module. KEGG identifiers were used to map both datasets to known metabolic and signaling pathways. The analysis applied the hypergeometric test for overrepresentation of genes and metabolites, combined with relative-betweenness centrality for pathway topology analysis, producing a combined pathway impact score. Pathways with a *P < 0.05* and impact scores > 0.1 were considered significantly enriched.

### Mouse eyecup flatmount dissection and morphology analysis

Mouse eyes were enucleated following euthanasia, and the anterior segment, lens, and neural retina were carefully removed to isolate the posterior eyecup containing the RPE-choroid-sclera complex. Four radial incisions were made to flatten the eyecup, which was then fixed in 4% PFA for 20 min at RT. Eyecups were then washed with PBS and blocked in 2% BSA, 0.1% Triton X-100 for 1 h at room temperature (RT). Samples were incubated overnight at 4°C with a primary antibody against ZO-1 (rabbit, Invitrogen 61-7300, 1:100). Following washes with PBS, samples were incubated with a fluorescently-labeled secondary antibody. Nuclei were counterstained with Hoechst 33342 (Thermo), and sections were mounted using ProLong Gold Antifade (Thermo). Images were acquired on a Zeiss LSM-900 microscope with Airyscan 2 using 20× magnification. Each flatmount was subdivided into central, mid-peripheral, and peripheral regions based on radial distance from the optic nerve head. Quantitative morphometric analysis of RPE cell shape parameters was performed using the REShAPE pipeline as previously published^26^.

### Two-photon excitation

TPE imaging and fluorescence lifetime data acquisition in mice were done as previously described^28^. The imaging instrument was based on the Leica TCS SP8 Falcon architecture. A custom, tunable light source consisting of a Ti:sapphire laser (Vision S, Coherent) and a pulse selection system was used to generate 780 nm, 4–80 MHz pulsing IR light. A custom periscope objective was used to measure fluorescence from distinct depths in the retina, namely sub-retinal space at the apical side of the RPE and inner retina. During imaging, an anesthetized mouse was surrounded by a heated pad and placed on a mechanical stage with its eye covered with GenTeal gel and a thin 3.2 mm diameter, 0 diopter contact lens (Cantor and Nissel). Fluorescence was measured as pixel mean gray value. Comparison of retinal fluorophores between mouse models was based on spectral detection with channels set to 400-660 nm to measure total fluorescence, 400-550 nm to measure fluorescence from retinyl esters, and 580-680 detecting fluorescence from retinal condensation products. Arbitrary color scale for FLIM imaging was assigned based on the phasor approach^28^, briefly phasor FLIM analyses start by displaying the fluorescence lifetime data from each imaging pixel as a point on the 2D graph, and then assigning arbitrary color to selected clusters of phasor points. These procedures were followed by the interpretation of the corresponding phasor FLIM image. FLIM analyses were performed using Leica LAS X FLIM Version 3.5.5 software.

### Transmission electron microscopy

Enucleated eyes were fixed overnight at 4 °C by immersion in 2% paraformaldehyde, 2.5% glutaraldehyde, and 5% CaCl_2_ in 0.1 M cacodylate buffer. After removing the anterior segment under a dissecting microscope, eyecups were processed for Epon embedding as previously described^108^. Ultra-thin sections (~85 nm) were cut using a diamond knife, stained with uranyl acetate and lead citrate, and examined using a Tecnai G2 Spirit BioTWIN transmission electron microscope operated at 60 kV.

### Lipidomic analysis

#### Lipid extraction

Lipid extractions were performed according to the methodology of Bligh and Dyer^109^. In brief, the tissue was homogenized in 200 μL water, transferred to a glass vial, and 750 μL 1:2 (v/v) CHCl_3_: MeOH was added and vortexed. Then 250 μL CHCl_3_ was added and vortexed. Finally, 250 μL ddH_2_O was added and vortexed. The samples were centrifuged at 3000 rpm for 5 min at 4 °C. The lower phase was transferred to a new glass vial, dried under nitrogen, and stored at −20 °C until subsequent lipid analysis.

#### LC-MS/MS

Separation of lipids was performed on an Accucore C30 column (2.6 μm, 2.1 mm × 150 mm, Thermo Scientific). The Q Exactive MS was operated in a full MS scan mode (resolution 70,000 at m/z 200) followed by ddMS2 (17,500 resolution) in both positive and negative modes. The AGC target value was set at 1E6 and 1E5 for the MS and MS/MS scans, respectively. The maximum injection time was 200 ms for MS and 50 ms for MS/MS. HCD was performed with a stepped collision energy of 30 ± 10% for negative and 25% and 30% for positive ion mode with an isolation window of 1.5 Da.

#### Data analysis and post-processing

Data were analyzed with LipidSearch 4.2.21 software. Only peaks with molecular identification grade: A or B were accepted (A: lipid class and fatty acids completely identified or B: lipid class and some fatty acids identified). The relative abundance of each lipid species was obtained by normalization to the total lipid intensity. Significantly changed lipid species (FC > 1.5. *P* < 0.05) were submitted to Lipid Ontology (LION) for lipid ontology analysis. Data visualization was performed using Prism 7 software (GraphPad Software, Inc.).

#### FA analysis

Separation of PUFAs was achieved on an Acquity UPLC^®^ BEH C18 column (1.7 μm, 2.1 mm × 100 mm, Waters Corporation). The Q Exactive MS was operated in a full MS scan mode (resolution 70,000 at m/z 200) in negative mode. For the compounds of interest, a scan range of m/z 250–800 was chosen. The identification of fatty acids was based on retention time and formula.

### Plasma membrane fractionation

Plasma membrane-enriched fractions were isolated from ARPE-19 cells grown in 15 cm dishes using the Minute^™^ Plasma Membrane/Protein Isolation and Cell Fractionation Kit (Invent Biotechnologies, cat. SM-005) according to the manufacturer’s instructions. Briefly, cells were trypsinized and pelleted by centrifugation at 500 ×*g* for 5 min at 4 °C. Cell pellets were resuspended in Buffer A supplemented with protease inhibitors and homogenized using the provided filter cartridge system. Sequential centrifugation steps were performed to separate cytosolic, organelle, and plasma membrane fractions. The final plasma membrane pellet was resuspended in PBS. Fraction purity was verified by immunoblotting using Na^+^/K^+^-ATPase as a plasma membrane marker and α-tubulin, PCNA, and TOM20 as cytosolic, nuclear, and mitochondrial/organelle markers, respectively. Plasma membrane fractions were subsequently used for global lipidomic analysis and subjected to C-Laurdan fluorescence spectroscopy to assess membrane order.

### Immunoblotting

Cells were harvested and lysed in RIPA buffer (50 mM Tris-HCl pH 7.4, 150 mM NaCl, 1% NP-40, 0.5% sodium deoxycholate, 0.1% SDS) supplemented with protease and phosphatase inhibitor cocktails (Roche). Protein concentrations were determined using the BCA assay (Thermo Fisher). Equal amounts of protein (5 μg) were mixed with 4× Laemmli sample buffer containing 10% β-mercaptoethanol, boiled at 95 °C for 5 min, and separated by SDS-PAGE on 4–12% gradient gels (Invitrogen). Proteins were transferred to PVDF membranes (Millipore) using the eBlot^™^ rapid transfer system (Genscript). Membranes were briefly stained with Ponceau S to verify uniform protein transfer and total protein loading before blocking and antibody incubation.

Membranes were blocked in 5% non-fat dry milk in PBS-T (20 mM Tris-HCl pH 7.6, 150 mM NaCl, 0.1% Tween-20) for 1 h at room temperature and incubated overnight at 4 °C with primary antibodies diluted in blocking buffer. Following three washes with PBS-T, membranes were incubated with HRP-conjugated secondary antibodies for 1 h at room temperature. Protein bands were visualized using enhanced chemiluminescence (ECL, Thermo Fisher) and imaged on a ChemiDoc system (Bio-Rad). Densitometric quantification was performed using ImageJ.

### C-Laurdan spectroscopy

Plasma membrane fractions were obtained from ARPE-19 cells grown on 15 cm plates and analyzed as previously described^110^. Briefly, samples were stained with 1 μm C-Laurdan (Tocris Bioscience) through addition of a 1 mM stock solution in DMSO. Samples were incubated with the dye for 30 min on ice. Fluorescence emission spectra were acquired on a Cary Eclipse Fluorescence spectrometer equipped a Peltier temperature controller maintained at 37 °C. Samples were excited at 355 nm with a 10 nm slit width and fluorescence spectra were acquired with a 1.5 nm slit width.

### Quantitative real-time PCR (qPCR)

Total RNA was extracted from differentiated ARPE-19 cells using the RNeasy Plus Mini Kit (Qiagen) following the manufacturer’s instructions. Complementary DNA (cDNA) was synthesized from total RNA using the SuperScript^™^ VILO cDNA Synthesis Kit (Thermo Fisher Scientific) following the manufacturer’s protocol. Quantitative PCR was performed using iTaq^™^ Universal SYBR^®^ Green Supermix (Bio-Rad) on a CFX384 Real-Time PCR Detection System (Bio-Rad). Reactions were run in technical triplicates under standard cycling conditions. Relative gene expression was calculated using the ΔΔCt method with normalization to *TBP* as the internal control. Primer sequences for target genes are listed in Table S2.

### Immunofluorescence

Cells were seeded in 12-well dishes containing laminin-coated coverslips and left to adhere overnight. After 3 days (ARPE-19) or 6 days (differentiated ARPE-19) in serum-depleted medium, cells were washed with PBS to remove the medium and fixed with 4% PFA for 15 min at RT. Next, cells were washed with PBS and permeabilized with 0.1% Triton X-100 for 10 min on ice. Cells were then washed with PBS and blocked with 5% BSA for 1 h at RT to minimize nonspecific binding, followed by incubation with primary antibodies in 5% BSA overnight at 4°C. Following three washes with PBS, cells were incubated with fluorescently-labeled secondary antibodies diluted in 5% BSA for 1 h at RT. Following three washes with PBS, nuclei were counterstained with Hoechst 33342 (Thermo), and coverslips were mounted using ProLong Gold Antifade (Thermo). Images were acquired on a Zeiss LSM-900 microscope with Airyscan 2 using 20× and 40× water immersion. The same brightness/contrast profile was applied to all images within the same experiment. ImageJ imaging software was used for image analysis.

### Flow cytometry

Flow cytometry analyses were performed using a WOLF^®^ G2 cell sorter (NanoCellect Biomedical). Cells were harvested by gentle dissociation, washed in PBS, and resuspended in PBS sorting buffer (Ca^2+^- and Mg^2+^-free PBS supplemented with 1% BSA, 1 mM EDTA, and 25 mM HEPES (pH 7.0)).

For surface staining, non-permeabilized cells were first blocked in flow cytometry buffer containing 5% BSA to reduce nonspecific binding, followed by incubation with a primary antibody against LAMP2 (1:100) for 30 min at RT. Cells were washed and then incubated with a fluorophore-conjugated secondary antibody for 30 min at RT. After additional washes, cells were resuspended in sorting buffer and analyze by flow cytometry.

Phagocytosis assays were performed following a previously published protocol^61^. Briefly, cells were incubated with FITC-labeled POS (InVision BioResources) for 0.5, 2, and 5 hours at 37°C. Cells were then rinsed with PBS and incubated with 0.05% trypsin-EDTA for 7 minutes to detach cells and release bound POS. Samples were resuspended in sorting buffer and analyzed by flow cytometry.

### Real-time Annexin V apoptosis monitoring

Phosphatidylserine (PS) exposure and apoptotic activation in live differentiated ARPE-19 cells were monitored using the RealTime-Glo^™^ Annexin V Apoptosis and Necrosis Assay (Promega, cat. JA1011), according to the manufacturer’s instructions. Briefly, differentiated ARPE-19 cells were transfected with either scrambled control or *ELOVL2*-targeting siRNA and seeded in 96-well plates. After 6 days of maintenance in serum-depleted, B27-supplemented medium, the RealTime-Glo^™^ Detection Reagent was prepared and added directly to the culture medium. Luminescence and fluorescence signals were measured every hour for 29 hours using a plate reader. H_2_O_2_-treated control cells (500 μM) were included as a positive control for apoptosis. Data were normalized to baseline luminescence and fluorescence (t = 0) and plotted.

### Endpoint fluorescent Annexin V assay

To validate real-time measurements of PS externalization, an endpoint fluorescent Annexin V assay was performed in parallel. Differentiated ARPE-19 (control and *ELOVL2*^KD^) cells were incubated with Invitrogen^™^ Annexin V Conjugates for Apoptosis Detection (Alexa Fluor 555) according to the manufacturer’s instructions. Cells were imaged using a Zeiss LSM-900 microscope with Airyscan 2 using 20× magnification and Annexin V puncta were quantified using ImageJ. H_2_O_2_-treated (1 mM) cells were included as a positive control for apoptosis.

### Lysosome distribution and positioning analysis

For quantitative analysis of lysosome positioning, we used the radial distribution method as previously described^111^. The distance from the cell nucleus to each LAMP1-labeled lysosome was calculated using ImageJ with a radial profiling plugin. Cells were subdivided into concentric zones from the nucleus to the cell periphery (perinuclear 0-30% radius, peripheral 30-60%). The proportion of lysosomes within each zone was calculated and compared between wild-type and *ELOVL2*^KD^ cells. For quantitative analysis of lysosome positioning, we used the radial distribution method as previously described^111^. The distance from the cell nucleus to each LAMP1-labelled lysosome was calculated using ImageJ with a radial profiling plugin. Cells were subdivided into concentric zones from the nucleus to the cell periphery (perinuclear 0-30% radius, peripheral 30-60%). The proportion of lysosomes within each zone was calculated and compared between wild-type and *ELOVL2*^KD^ cells.

### Proximity ligation assay

Reagents for the PLA of differentiated ARPE-19 cells were purchased and used using instructions from Sigma-Aldrich (DUO92004 and DUO92002). Briefly, cells were fixed on coverslips and prepared for staining with primary antibodies as described above. Anti-ZO-1, anti-LAMP2, and anti-IgG were used at 1:100 dilution. Anti-IgG was used as a negative control. Instead of fluorescently tagged secondary antibodies, specimens were incubated with anti-mouse and anti-rabbit oligonucleotide-tethered secondary antibodies. Close proximity of target antigens allows their respective secondary antibody nucleotide sequences to hybridize. Following a 30-min 37°C incubation with ligase and additional oligonucleotides, a closed DNA circle was formed. A subsequent step involving polymerase-driven rolling circle amplification incorporated fluorescently labeled nucleotides. Fluorescent nucleotides used in this experiment fluoresce in the red channel upon excitation. Fluorescent spots appear at sites of ZO-1/LAMP2 protein-protein interactions when using primary antibodies and were evaluated using confocal microscopy and quantified using ImageJ. Data were normalized by applying the same brightness/contrast profile and threshold values. ImageJ’s Analyze Particles feature was used to quantify PLA spots over 10 fields of view for each condition (n > 30). Raw values were then converted to fold change with respect to the appropriate control.

### β-hexosaminidase release assay

Extracellular release of β-hexosaminidase was quantified as a marker of lysosomal exocytosis. Cells were plated in 12-well plates and cultured in phenol-red free media for 6 days. Culture supernatants were collected and centrifuged at 300 x*g* for 10 min and 2000 x*g* for 10 min to remove cell debris. The clarified supernatants were then concentrated using Amicon Ultra centrifugal filters (50 kDa cutoff at 3,000 x*g* for 5 min, followed by 3 kDa cutoff at 14,000 x*g* for 30 min). Cells were then washed once with ice-cold PBS and lysed in RIPA buffer supplemented with protease inhibitor cocktail. Both supernatants and lysates were incubated with 4-methylumbelliferyl N-acetyl-β-D-glucosaminide substrate (2 mM in sodium-citrate-phosphate buffer, pH 4.5) for 30 min at RT. The reaction was terminated by adding 0.2 M glycine, pH 10. Fluorescence intensity was measured at excitation/emission wavelengths of 365/450 nm using a microplate reader. Enzyme release was quantified as the ratio of β-hexosaminidase activity in the supernatant to that in the corresponding lysate, providing a measure of relative extracellular enzyme secretion.

### Lattice light-sheet microscopy of live cells

Four-dimensional imaging was performed using a custom-built lattice light-sheet microscope developed by the Eric Betzig laboratory at HHMI Janelia Research Campus and UC Berkeley^53^. The system was equipped with a 0.6 NA excitation objective (Thorlabs TL20X-MPL) and a 1.0 NA detection objective (Zeiss W Plan-Apochromat 20×/1.0, model #421452-9800) and a Hamamatsu ORCA-Fusion BT sCMOS camera.

For live-cell labeling, cells were incubated with 50 nM LysoTracker Green for 30 min at 37 °C. Following LysoTracker labeling, cells were washed and incubated with 5 μg/mL CellMask Deep Red plasma membrane stain for 5 min at 37 °C. After staining, cells were washed and imaged immediately in phenol red-free imaging medium.

Excitation was provided by 488-nm and 642-nm lasers to image LysoTracker Green and CellMask Deep Red, respectively. Illumination was generated using a multiple Bessel beam in a lattice light-sheet pattern with a numerical aperture range of NA_min_ = 0.35 to NA_max_ = 0.40. During imaging, samples were maintained at 37 °C in a humidified atmosphere containing 5% CO_2_. Raw lattice light-sheet microscopy data were preprocessed using the LiveLattice pipeline^54^, including camera background subtraction, photobleaching correction, sample-scan deconvolution, deskewing, and rotation into a standard Cartesian coordinate system. Each preprocessed 3D volume comprised 864 × 1536 × 202 voxels (x, y, z) with an isotropic voxel size of 0.111 μm. Time-lapse imaging was performed at 5.6-s intervals for a total of 60 frames.

### Intravitreal injections

Intravitreal injections were performed as previously described^11^ as part of a separate study. For the present work, RPE eyecups from these animals were collected and analyzed to assess lipidomic and cellular outcomes.

## Supplementary Material

Supplement 1 Fig. S1. Two-photon fluorescence lifetime imaging reveals sub-retinal deposits in *Elovl2*^C234W^ mice.
**a.** Relative abundances of LC- and VLC-PUFAs in eyecups from 18-month-old wild-type mice relative to 3-month-old wild-type mice (*n* = 5 per group).
**b.** Representative *in vivo* two-photon fundus images from wild-type and *Elovl2*^C234W^ mice obtained with 760 nm excitation. Top: grayscale intensity images. Middle: FLIM semicircle phasor plots. Clusters of phasor points are color mapped from blue to red, such that red represents highest pixel density. Bottom: FLIM phasor images based on colors assigned in corresponding semicircle phasor plots.
**c.** Representative grayscale two-photon autofluorescence images acquired across 400-660 nm, 400-550 nm, and 580-680 nm wavelength ranges. Statistical significance was determined using unpaired t-tests (**a**). *P < 0.05, **P < 0.01, ***P < 0.001, and ****P < 0.0001. Source numerical data are available in Source Data.
Fig S2. Disrupting LC-PUFA synthesis drives ultrastructural defects and lipid remodeling.
**a.** Relative abundances of LC- and VLC-PUFAs in eyecups from 18-month-old *Elovl2*^C234W^ mice relative to age-matched wild-type mice (*n* = 3 per group).
**b-c.** Heatmap (**b**) and relative abundances (**c**) of lipid classes in eyecups from 18-month-old *Elovl2*^C234W^ mice relative to age-matched wild-type (*n =* 2 per group). Statistical significance was determined using unpaired t-tests (**a**, **b**, **c**). *P < 0.05, **P < 0.01, ***P < 0.001, and ****P < 0.0001. Source numerical data are available in Source Data.
Fig S3. ELOVL2 loss remodels plasma membrane lipidome and induces membrane stress.
**a.** qPCR analysis of *RPE65* and *ELOVL2* expression in differentiated control and *ELOVL2*^KD^ cells (*n* = 4 per group). The dashed line indicates expression level in undifferentiated ARPE-19 cells. Data are presented as mean ± SEM.
**b-c.** Relative abundances of LC- and VLC-PUFAs (**b**) and lipid classes (**c**) in ELOVL2^KD^ cells compared to control cells (n = 4 per group).
**d.** Immunoblot analysis of nuclear, cytoplasmic, organelle, and plasma membrane fractions. Fraction purity was assessed using marker proteins: Na^+^/K^+^-ATPase for plasma membrane, α-tubulin for cytoplasm, and TOM20 for mitochondria/organelles.
**e.** Heatmap of ceramide saturation profiles (SFA, MUFA, PUFA) in eyecups from 18-month-old wild-type mice relative to 3-month-old wild-type (*n* = 5 per group).
**f.** Real-time measurement of phosphatidylserine externalization (Annexin V binding, solid lines) and apoptosis activation (dashed lines) in cells treated with 500 μM H_2_O_2_ to induce apoptosis (*n* = 3 for 0 μM H_2_O_2_ and *n* = 4 for 500 μM H_2_O_2_).
**g-h.** Representative confocal images of Annexin V (red) (**g**) and caspase-9 (green) and cleaved caspase-3 (red) (**h**) in cells treated with 500 μM H_2_O_2_. Annexin V staining was assessed after 1 hour of treatment, and caspase activation was assessed 6 hours after treatment. Scale = 10 μm. Statistical significance was determined using unpaired t-tests (**a**, **b**, **c**, **e**) or two-way ANOVA (**f**). *P < 0.05, **P < 0.01, ***P < 0.001, and ****P < 0.0001. Source numerical data are available in Source Data.
Fig. S4. *ELOVL2* deficiency alters lysosomal dynamics and lysosome-plasma membrane fusion.
**a.** Representative confocal images of LAMP1-positive lysosomes (green) in control and *ELOVL2*^KD^ cells, overlaid with concentric radial shells used to quantify lysosome distribution relative to the cell size. Scale = 10 μm.
**b-c.** Quantification of lysosome number (**b**) and size (**c**). Data are presented as replicate mean ± SEM (closed circles; 3 per group) and fields of view (open circles; >7 per replicate).
**d.** Representative confocal images of ZO-1 (green) and LAMP2 (red) in control and *ELOVL2*^KD^ cells. Scale = 10 μm.
**e.** Left: schematic illustration of lysosome-plasma membrane fusion as detected by cell-surface exposure of luminal LAMP2 epitope. Right: flow cytometry analysis of LAMP2-positive non-permeabilized cells (*n* = 8 per group).
**f.** β-hexosaminidase activity in the culture supernatant (SN, left) relative to that in the corresponding cell lysate (L, right), normalized to protein concentration. Data are presented as mean ± SEM (*n* = 7 in control and *n* = 8 in *ELOVL2*^KD^).
**g.** Left: representative images of 1D4-positive phagosomes (green) in RPE flatmounts stained for ZO-1 (red) from 18-month-old wild-type and *Elovl2*^C234W^ mice and imaged at different times after light onset (ZT1, ZT4, ZT8). Scale = 20 μm. Middle-right: quantification of phagosome count (middle) and size (right). Data are presented as mean ± SEM (*n* = 3 animals per group).
**h.** *Left*: flow cytometry-based POS phagocytosis assay of control and *ELOVL2*^KD^ cells incubated with FITC-labeled POS. *Right*: POS uptake (% FITC^+^ cells) was quantified at 0.5, 2 and 5 hours after incubation. Data are presented as mean ± SEM (*n* = 2 per group at t = 0 and 0.5, and *n* = 3 per group at t = 2 and 5).
**i.** Left: Immunoblot showing LC3-I and LC3-II levels in control and *ELOVL2*^KO^ iPSC-RPE. Ponceau S staining is shown as a total protein loading control. Right: quantification of autophagic flux expressed as the LC3-II/LC3-I ratio. Data are presented as mean ± SEM (*n* = 5 for control and *n* = 4 for *ELOVL2*^KO^).
**j.** qPCR analysis of *mTOR* expression in control and *ELOVL2*^KD^ cells. Data are presented as mean ± SEM (*n* = 3 in control and *n* = 4 in *ELOVL2*^KD^). Statistical significance was determined using unpaired t-tests (**b, c, e, f, i, j**) or two-way ANOVA (**g, h**). *P < 0.05, **P < 0.01, ***P < 0.001, and ****P < 0.0001. Source numerical data are available in Source Data.
Fig. S5. *ELOVL2* enzymatic activity is necessary to maintain lipid homeostasis.
**a.** Relative abundances of lipid classes in *ELOVL2*^KO^ iPSC-RPE compared to control cells (n = 6 per group).
**b.** Relative abundances of ceramide lipid species in eyecups from 24:5n-3-supplemented 16-month-old mice relative to eyecups from vehicle-injected mice (n = 3 per group). Data are presented as mean ± SEM. Statistical significance was determined using unpaired t-tests (**a, b**). *P < 0.05, **P < 0.01, ***P < 0.001, and ****P < 0.0001. Source numerical data are available in Source Data.

## Data Availability

All data supporting the findings of this study are available in the main text, extended data figures and supplementary materials. Bulk RNA sequencing data generated in this study will be deposited in the NCBI Gene Expression Omnibus (GEO). *Elovl2*^C234W^ animals are available to interested researchers through a material transfer agreement with University of California, Irvine.

## References

[1] Lakkaraju A. The cell biology of the retinal pigment epithelium. Progress in Retinal and Eye Research 78, 100846 (2020). 10.1016/j.preteyeres.2020.100846 PMC8941496 32105772

[2] Handa J. T. A systems biology approach towards understanding and treating non-neovascular age-related macular degeneration. Nat Commun 10, 3347 (2019). 31350409 10.1038/s41467-019-11262-1 PMC6659646

[3] Tan L. X., Germer C. J., La Cunza N. & Lakkaraju A. Complement activation, lipid metabolism, and mitochondrial injury: Converging pathways in age-related macular degeneration. Redox Biol 37, 101781 (2020). 33162377 10.1016/j.redox.2020.101781 PMC7767764

[4] Sunshine H. & Iruela-Arispe M. L. Membrane Lipids and Cell Signaling. Curr Opin Lipidol 28, 408–413 (2017). 28692598 10.1097/MOL.0000000000000443 PMC5776726

[5] Harayama T. & Riezman H. Understanding the diversity of membrane lipid composition. Nat Rev Mol Cell Biol 19, 281–296 (2018). 29410529 10.1038/nrm.2017.138

[6] Datta S., Cano M., Ebrahimi K., Wang L. & Handa J. T. The impact of oxidative stress and inflammation on RPE degeneration in non-neovascular AMD. Progress in Retinal and Eye Research 60, 201–218 (2017). 28336424 10.1016/j.preteyeres.2017.03.002 PMC5600827

[7] Garagnani P. Methylation of ELOVL2 gene as a new epigenetic marker of age. Aging Cell 11, 1132–1134 (2012). 23061750 10.1111/acel.12005

[8] Spólnicka M. DNA methylation in ELOVL2 and C1orf132 correctly predicted chronological age of individuals from three disease groups. Int J Legal Med 132, 1–11 (2018). 28725932 10.1007/s00414-017-1636-0 PMC5748441

[9] Bacalini M. G. Systemic Age-Associated DNA Hypermethylation of ELOVL2 Gene: In Vivo and In Vitro Evidences of a Cell Replication Process. J Gerontol A Biol Sci Med Sci 72, 1015–1023 (2017). 27672102 10.1093/gerona/glw185 PMC5861890

[10] Chen D. The lipid elongation enzyme ELOVL2 is a molecular regulator of aging in the retina. Aging Cell 19, e13100 (2020). 31943697 10.1111/acel.13100 PMC6996962

[11] Gao F. Retinal polyunsaturated fatty acid supplementation reverses aging-related vision decline in mice. Science Translational Medicine 17, eads5769 (2025). 40991728 10.1126/scitranslmed.ads5769 PMC13033240

[12] Zhao X. Lipidomic profiling reveals age-dependent changes in complex plasma membrane lipids that regulate neural stem cell aging. 2022.08.18.503095 Preprint at 10.1101/2022.08.18.503095 (2022). PMC13418745 42525742

[13] Mota-Martorell N. Selective brain regional changes in lipid profile with human aging. GeroScience 44, 763–783 (2022). 35149960 10.1007/s11357-022-00527-1 PMC9135931

[14] Dai Y., Tang H. & Pang S. The Crucial Roles of Phospholipids in Aging and Lifespan Regulation. Front Physiol 12, 775648 (2021). 34887779 10.3389/fphys.2021.775648 PMC8650052

[15] Skowronska-Krawczyk D. & Budin I. Aging membranes: unexplored functions for lipids in the lifespan of the central nervous system. Exp Gerontol 131, 110817 (2020). 31862420 10.1016/j.exger.2019.110817 PMC7877915

[16] Butler J. M., Supharattanasitthi W., Yang Y. C. & Paraoan L. RNA-seq analysis of ageing human retinal pigment epithelium: Unexpected up-regulation of visual cycle gene transcription. J Cell Mol Med 25, 5572–5585 (2021). 33934486 10.1111/jcmm.16569 PMC8184696

[17] Thomas P. D. PANTHER: Making genome-scale phylogenetics accessible to all. Protein Sci 31, 8–22 (2022). 34717010 10.1002/pro.4218 PMC8740835

[18] Sangiovanni J. P. {omega}-3 Long-chain polyunsaturated fatty acid intake and 12-y incidence of neovascular age-related macular degeneration and central geographic atrophy: AREDS report 30, a prospective cohort study from the Age-Related Eye Disease Study. Am J Clin Nutr 90, 1601–1607 (2009). 19812176 10.3945/ajcn.2009.27594 PMC2777471

[19] van Leeuwen E. M. A new perspective on lipid research in age-related macular degeneration. Progress in Retinal and Eye Research 67, 56–86 (2018). 29729972 10.1016/j.preteyeres.2018.04.006

[20] Liu A., Chang J., Lin Y., Shen Z. & Bernstein P. S. Long-chain and very long-chain polyunsaturated fatty acids in ocular aging and age-related macular degeneration. J Lipid Res 51, 3217–3229 (2010). 20688753 10.1194/jlr.M007518 PMC2952562

[21] Subramanian A. Gene set enrichment analysis: A knowledge-based approach for interpreting genome-wide expression profiles. Proceedings of the National Academy of Sciences 102, 15545–15550 (2005). 10.1073/pnas.0506580102 PMC1239896 16199517

[22] Zhou M. Role of Epithelial-Mesenchymal Transition in Retinal Pigment Epithelium Dysfunction. Front Cell Dev Biol 8, 501 (2020). 32671066 10.3389/fcell.2020.00501 PMC7329994

[23] Geerlings M. J., de Jong E. K. & den Hollander A. I. The complement system in age-related macular degeneration: A review of rare genetic variants and implications for personalized treatment. Mol Immunol 84, 65–76 (2017). 27939104 10.1016/j.molimm.2016.11.016 PMC5380947

[24] Armento A., Ueffing M. & Clark S. J. The complement system in age-related macular degeneration. Cell Mol Life Sci 78, 4487–4505 (2021). 33751148 10.1007/s00018-021-03796-9 PMC8195907

[25] Wilke G. A. & Apte R. S. Complement regulation in the eye: implications for age-related macular degeneration. J Clin Invest 134, (2024). 10.1172/JCI178296 PMC11060743 38690727

[26] Ortolan D. Single-cell–resolution map of human retinal pigment epithelium helps discover subpopulations with differential disease sensitivity. Proceedings of the National Academy of Sciences 119, e2117553119 (2022). 10.1073/pnas.2117553119 PMC9171647 35522714

[27] Kim Y.-K. Morphometric Analysis of Retinal Pigment Epithelial Cells From C57BL/6J Mice During Aging. Invest. Ophthalmol. Vis. Sci. 62, 32 (2021). 10.1167/iovs.62.2.32 PMC7910641 33616620

[28] Palczewska G. Noninvasive two-photon optical biopsy of retinal fluorophores. Proceedings of the National Academy of Sciences 117, 22532–22543 (2020). 10.1073/pnas.2007527117 PMC7486747 32848058

[29] Palczewska G. Noninvasive multiphoton fluorescence microscopy resolves retinol and retinal condensation products in mouse eyes. Nat Med 16, 1444–1449 (2010). 21076393 10.1038/nm.2260 PMC3057900

[30] Mishima H. & Kondo K. Ultrastructure of age changes in the basal infoldings of aged mouse retinal pigment epithelium. Experimental Eye Research 33, 75–84 (1981). 7250233 10.1016/s0014-4835(81)80083-8

[31] Bonilha V. L. Age and disease-related structural changes in the retinal pigment epithelium. Clinical Ophthalmology 2, 413–424 (2008). 19668732 10.2147/opth.s2151 PMC2693982

[32] Hayes M. J. Remodeling of the Basal Labyrinth of Retinal Pigment Epithelial Cells With Osmotic Challenge, Age, and Disease. Invest Ophthalmol Vis Sci 60, 2515–2524 (2019). 31194861 10.1167/iovs.19-26784 PMC6564051

[33] Pilgrim M. G. Subretinal Pigment Epithelial Deposition of Drusen Components Including Hydroxyapatite in a Primary Cell Culture Model. Invest Ophthalmol Vis Sci 58, 708–719 (2017). 28146236 10.1167/iovs.16-21060 PMC5295770

[34] Chen S. Ultrahigh Resolution OCT Markers of Normal Aging and Early Age-related Macular Degeneration. Ophthalmol Sci 3, 100277 (2023). 36970115 10.1016/j.xops.2023.100277 PMC10034509

[35] Molenaar M. R. LION/web: a web-based ontology enrichment tool for lipidomic data analysis. Gigascience 8, giz061 (2019). 31141612 10.1093/gigascience/giz061 PMC6541037

[36] Alonso A. & Goñi F. M. The Physical Properties of Ceramides in Membranes. Annu Rev Biophys 47, 633–654 (2018). 29618220 10.1146/annurev-biophys-070317-033309

[37] Kaltenegger M. Intrinsic lipid curvatures of mammalian plasma membrane outer leaflet lipids and ceramides. Biochim Biophys Acta Biomembr 1863, 183709 (2021). 34332987 10.1016/j.bbamem.2021.183709

[38] Pang Z. MetaboAnalyst 6.0: towards a unified platform for metabolomics data processing, analysis and interpretation. Nucleic Acids Res 52, W398–W406 (2024). 38587201 10.1093/nar/gkae253 PMC11223798

[39] Hazim R. A., Volland S., Yen A., Burgess B. L. & Williams D. S. Rapid differentiation of the human RPE cell line, ARPE-19, induced by nicotinamide. Exp Eye Res 179, 18–24 (2019). 30336127 10.1016/j.exer.2018.10.009 PMC6360117

[40] López-Montero I., Monroy F., Vélez M. & Devaux P. F. Ceramide: From lateral segregation to mechanical stress. Biochimica et Biophysica Acta (BBA) - Biomembranes 1798, 1348–1356 (2010). 20026045 10.1016/j.bbamem.2009.12.007

[41] Dawaliby R. Phosphatidylethanolamine Is a Key Regulator of Membrane Fluidity in Eukaryotic Cells*. Journal of Biological Chemistry 291, 3658–3667 (2016). 26663081 10.1074/jbc.M115.706523 PMC4751403

[42] Levitan I. Evaluating membrane structure by Laurdan imaging: Disruption of lipid packing by oxidized lipids. Curr Top Membr 88, 235–256 (2021). 34862028 10.1016/bs.ctm.2021.10.003 PMC8929669

[43] Veiga M. P., Arrondo J. L. R., Goñi F. M. & Alonso A. Ceramides in Phospholipid Membranes: Effects on Bilayer Stability and Transition to Nonlamellar Phases. Biophysical Journal 76, 342–350 (1999). 9876146 10.1016/S0006-3495(99)77201-2 PMC1302523

[44] Sot J., Goñi F. M. & Alonso A. Molecular associations and surface-active properties of short- and long-*N*-acyl chain ceramides. Biochimica et Biophysica Acta (BBA) - Biomembranes 1711, 12–19 (2005). 15904658 10.1016/j.bbamem.2005.02.014

[45] Contreras F.-X., Basañez G., Alonso A., Herrmann A. & Goñi F. M. Asymmetric addition of ceramides but not dihydroceramides promotes transbilayer (flip-flop) lipid motion in membranes. Biophys J 88, 348–359 (2005). 15465865 10.1529/biophysj.104.050690 PMC1305011

[46] Pilátová M. B., Solárová Z., Mezencev R. & Solár P. Ceramides and their roles in programmed cell death. Advances in Medical Sciences 68, 417–425 (2023). 37866204 10.1016/j.advms.2023.10.004

[47] Obeid L. M., Linardic C. M., Karolak L. A. & Hannun Y. A. Programmed Cell Death Induced by Ceramide. Science 259, 1769–1771 (1993). 8456305 10.1126/science.8456305

[48] Chen X. Hydrogen peroxide-induced oxidative damage and protective role of peroxiredoxin 6 protein via EGFR/ERK signaling pathway in RPE cells. Front Aging Neurosci 15, 1169211 (2023). 37529008 10.3389/fnagi.2023.1169211 PMC10388243

[49] Reddy A., Caler E. V. & Andrews N. W. Plasma Membrane Repair Is Mediated by Ca2+-Regulated Exocytosis of Lysosomes. Cell 106, 157–169 (2001). 11511344 10.1016/s0092-8674(01)00421-4

[50] Encarnação M. A Rab3a-dependent complex essential for lysosome positioning and plasma membrane repair. J Cell Biol 213, 631–640 (2016). 27325790 10.1083/jcb.201511093 PMC4915190

[51] Corrotte M. & Castro-Gomes T. Lysosomes and plasma membrane repair. Curr Top Membr 84, 1–16 (2019). 31610859 10.1016/bs.ctm.2019.08.001

[52] Zhao Q., Gao S. M. & Wang M. C. Molecular Mechanisms of Lysosome and Nucleus Communication. Trends in Biochemical Sciences 45, 978–991 (2020). 32624271 10.1016/j.tibs.2020.06.004 PMC7572682

[53] Fu T.-M. A Multimodal Adaptive Optical Microscope For In Vivo Imaging from Molecules to Organisms. 2025.06.02.657494 Preprint at 10.1101/2025.06.02.657494 (2025). PMC13310421 42174242

[54] Wang Z., Hakozaki H., McMahon G., Medina-Carbonero M. & Schöneberg J. LiveLattice: Real-time visualisation of tilted light-sheet microscopy data using a memory-efficient transformation algorithm. Journal of Microscopy 297, 123–134 (2025). 39360400 10.1111/jmi.13358 PMC11733850

[55] Andrews N. W. Detection of Lysosomal Exocytosis by Surface Exposure of Lamp1 Luminal Epitopes. Methods Mol Biol 1594, 205–211 (2017). 28456985 10.1007/978-1-4939-6934-0_13

[56] Castro-Gomes T., Corrotte M., Tam C. & Andrews N. W. Plasma Membrane Repair Is Regulated Extracellularly by Proteases Released from Lysosomes. PLOS ONE 11, e0152583 (2016). 27028538 10.1371/journal.pone.0152583 PMC4814109

[57] Sinha D. Lysosomes: regulators of autophagy in the retinal pigmented epithelium. Exp Eye Res 144, 46–53 (2016). 26321509 10.1016/j.exer.2015.08.018 PMC4698066

[58] Boya P., Kaarniranta K., Handa J. T. & Sinha D. Lysosomes in retinal health and disease. Trends Neurosci 46, 1067–1082 (2023). 37848361 10.1016/j.tins.2023.09.006 PMC10842632

[59] Vargas J. A. & Finnemann S. C. Probing Photoreceptor Outer Segment Phagocytosis by the RPE In Vivo: Models and Methodologies. Int J Mol Sci 23, 3661 (2022). 35409021 10.3390/ijms23073661 PMC8998817

[60] Lieffrig S. A., Gyimesi G., Mao Y. & Finnemann S. C. Clearance phagocytosis by the retinal pigment epithelium during photoreceptor outer segment renewal: Molecular mechanisms and relation to retinal inflammation. Immunol Rev 319, 81–99 (2023). 37555340 10.1111/imr.13264 PMC10615845

[61] Westenskow P. D. Using Flow Cytometry to Compare the Dynamics of Photoreceptor Outer Segment Phagocytosis in iPS-Derived RPE Cells. Invest Ophthalmol Vis Sci 53, 6282–6290 (2012). 22871841 10.1167/iovs.12-9721 PMC3444211

[62] Sharma R. Epithelial phenotype restoring drugs suppress macular degeneration phenotypes in an iPSC model. Nat Commun 12, 7293 (2021). 34911940 10.1038/s41467-021-27488-x PMC8674335

[63] Li H., Sharma R. & Bharti K. iPSC-derived retinal pigment epithelium: an in vitro platform to reproduce key cellular phenotypes and pathophysiology of retinal degenerative diseases. Stem Cells Transl Med 14, szae097 (2025). 39729520 10.1093/stcltm/szae097 PMC11954503

[64] Wahlin K. J. CRISPR Generated SIX6 and POU4F2 Reporters Allow Identification of Brain and Optic Transcriptional Differences in Human PSC-Derived Organoids. Front Cell Dev Biol 9, 764725 (2021). 34869356 10.3389/fcell.2021.764725 PMC8635054

[65] Sharma R., Bose D., Montford J., Ortolan D. & Bharti K. Triphasic developmentally guided protocol to generate retinal pigment epithelium from induced pluripotent stem cells. STAR Protocols 3, 101582 (2022). 35880133 10.1016/j.xpro.2022.101582 PMC9307589

[66] Tam C. Exocytosis of acid sphingomyelinase by wounded cells promotes endocytosis and plasma membrane repair. J Cell Biol 189, 1027–1038 (2010). 20530211 10.1083/jcb.201003053 PMC2886342

[67] Klionsky D. J. Guidelines for the use and interpretation of assays for monitoring autophagy (4th edition)1. Autophagy 17, 1–382 (2021). 33634751 10.1080/15548627.2020.1797280 PMC7996087

[68] Mitter S. K. Dysregulated autophagy in the RPE is associated with increased susceptibility to oxidative stress and AMD. Autophagy 10, 1989–2005 (2014). 25484094 10.4161/auto.36184 PMC4502658

[69] Golestaneh N., Chu Y., Xiao Y.-Y., Stoleru G. L. & Theos A. C. Dysfunctional autophagy in RPE, a contributing factor in age-related macular degeneration. Cell Death Dis 8, e2537–e2537 (2018). 10.1038/cddis.2016.453 PMC5386365 28055007

[70] Go Y.-M. MTOR-initiated metabolic switch and degeneration in the retinal pigment epithelium. The FASEB Journal 34, 12502–12520 (2020). 32721041 10.1096/fj.202000612R PMC7811279

[71] Anderson D. H. Local cellular sources of apolipoprotein E in the human retina and retinal pigmented epithelium: implications for the process of drusen formation. American Journal of Ophthalmology 131, 767–781 (2001). 11384575 10.1016/s0002-9394(00)00961-2

[72] Johnson L. V. Cell culture model that mimics drusen formation and triggers complement activation associated with age-related macular degeneration. Proceedings of the National Academy of Sciences 108, 18277–18282 (2011). 10.1073/pnas.1109703108 PMC3215058 21969589

[73] Glaser P. E. & Gross R. W. Plasmenylethanolamine facilitates rapid membrane fusion: a stopped-flow kinetic investigation correlating the propensity of a major plasma membrane constituent to adopt an HII phase with its ability to promote membrane fusion. Biochemistry 33, 5805–5812 (1994). 8180209 10.1021/bi00185a019

[74] Kaur G. Aberrant early endosome biogenesis mediates complement activation in the retinal pigment epithelium in models of macular degeneration. Proc Natl Acad Sci U S A 115, 9014–9019 (2018). 30126999 10.1073/pnas.1805039115 PMC6130344

[75] Toops K. A., Tan L. X., Jiang Z., Radu R. A. & Lakkaraju A. Cholesterol-mediated activation of acid sphingomyelinase disrupts autophagy in the retinal pigment epithelium. Mol Biol Cell 26, 1–14 (2015). 25378587 10.1091/mbc.E14-05-1028 PMC4279221

[76] Cunza N. L. Mitochondria-dependent phase separation of disease-relevant proteins drives pathological features of age-related macular degeneration. JCI Insight 6, (2021). 10.1172/jci.insight.142254 PMC8262309 33822768

[77] Lewandowski D. Inhibition of ceramide accumulation in AdipoR1−/− mice increases photoreceptor survival and improves vision. JCI Insight 7, e156301. 10.1172/jci.insight.156301 PMC8876453 35015730

[78] Lewandowski D. Restoring retinal polyunsaturated fatty acid balance and retina function by targeting ceramide in AdipoR1-deficient mice. Journal of Biological Chemistry 300, (2024). 10.1016/j.jbc.2024.107291 PMC11107370 38636661

[79] Zhang K. R. Oxidative stress induces lysosomal membrane permeabilization and ceramide accumulation in retinal pigment epithelial cells. Dis Model Mech 16, dmm050066 (2023). 37401371 10.1242/dmm.050066 PMC10399446

[80] Andrews N. W. Solving the secretory acid sphingomyelinase puzzle: Insights from lysosome-mediated parasite invasion and plasma membrane repair. Cell Microbiol 21, e13065 (2019). 31155842 10.1111/cmi.13065 PMC6842087

[81] Ramachandran P. V. Lysosomal Signaling Promotes Longevity through Adjusting Mitochondrial Activity. Dev Cell 48, 685–696.e5 (2019). 30713071 10.1016/j.devcel.2018.12.022 PMC6613828

[82] Savini M. Lysosome lipid signalling from the periphery to neurons regulates longevity. Nat Cell Biol 24, 906–916 (2022). 35681008 10.1038/s41556-022-00926-8 PMC9203275

[83] Zhang Q., Dang W. & Wang M. C. Lysosomes signal through the epigenome to regulate longevity across generations. Science 389, 1353–1360 (2025). 40997170 10.1126/science.adn8754 PMC12831228

[84] Li T. Y. Lysosomes mediate the mitochondrial UPR via mTORC1-dependent ATF4 phosphorylation. Cell Discov 9, 92 (2023). 37679337 10.1038/s41421-023-00589-1 PMC10484937

[85] Lawrence R. E. & Zoncu R. The lysosome as a cellular centre for signalling, metabolism and quality control. Nat Cell Biol 21, 133–142 (2019). 30602725 10.1038/s41556-018-0244-7

[86] Shin H. R. Lysosomal GPCR-like protein LYCHOS signals cholesterol sufficiency to mTORC1. Science 377, 1290–1298 (2022). 36007018 10.1126/science.abg6621 PMC10023259

[87] Guerrero-Navarro L., Jansen-Dürr P. & Cavinato M. Age-Related Lysosomal Dysfunctions. Cells 11, 1977 (2022). 35741106 10.3390/cells11121977 PMC9221958

[88] Rubinsztein D. C., Mariño G. & Kroemer G. Autophagy and Aging. Cell 146, 682–695 (2011). 21884931 10.1016/j.cell.2011.07.030

[89] Nixon R. A. The aging lysosome: An essential catalyst for late-onset neurodegenerative diseases. Biochimica et Biophysica Acta (BBA) - Proteins and Proteomics 1868, 140443 (2020). 32416272 10.1016/j.bbapap.2020.140443 PMC7388076

[90] Peng W., Minakaki G., Nguyen M. & Krainc D. Preserving Lysosomal Function in the Aging Brain: Insights from Neurodegeneration. Neurotherapeutics 16, 611–634 (2019). 31183763 10.1007/s13311-019-00742-3 PMC6694346

[91] Martinez-Lopez N., Athonvarangkul D. & Singh R. Autophagy and Aging. Adv Exp Med Biol 847, 73–87 (2015). 25916586 10.1007/978-1-4939-2404-2_3 PMC4644734

[92] Kim S.-H., Cho Y.-S. & Jung Y.-K. Failure of lysosomal acidification and endomembrane network in neurodegeneration. Exp Mol Med 57, 2418–2428 (2025). 41254240 10.1038/s12276-025-01579-x PMC12686521

[93] Karan G. Lipofuscin accumulation, abnormal electrophysiology, and photoreceptor degeneration in mutant ELOVL4 transgenic mice: a model for macular degeneration. Proc Natl Acad Sci U S A 102, 4164–4169 (2005). 15749821 10.1073/pnas.0407698102 PMC554798

[94] Agbaga M.-P. Role of Stargardt-3 macular dystrophy protein (ELOVL4) in the biosynthesis of very long chain fatty acids. Proc Natl Acad Sci U S A 105, 12843–12848 (2008). 18728184 10.1073/pnas.0802607105 PMC2525561

[95] Barabas P. Role of ELOVL4 and very long-chain polyunsaturated fatty acids in mouse models of Stargardt type 3 retinal degeneration. Proceedings of the National Academy of Sciences 110, 5181–5186 (2013). 10.1073/pnas.1214707110 PMC3612655 23479632

[96] Lewandowski D. Dynamic lipid turnover in photoreceptors and retinal pigment epithelium throughout life. Prog Retin Eye Res 89, 101037 (2022). 34971765 10.1016/j.preteyeres.2021.101037 PMC10361839

[97] Cell Culture in the Neurosciences. (Springer US, Boston, MA, 1985). doi:10.1007/978-1-4613-2473-7.

[98] Kuo H.-H. Negligible-Cost and Weekend-Free Chemically Defined Human iPSC Culture. Stem Cell Reports 14, 256–270 (2020). 31928950 10.1016/j.stemcr.2019.12.007 PMC7013200

[99] Brewer G. J., Torricelli J. R., Evege E. K. & Price P. J. Optimized survival of hippocampal neurons in B27-supplemented Neurobasal, a new serum-free medium combination. J Neurosci Res 35, 567–576 (1993). 8377226 10.1002/jnr.490350513

[100] Bhise N. S., Wahlin K. J., Zack D. J. & Green J. J. Evaluating the potential of poly(beta-amino ester) nanoparticles for reprogramming human fibroblasts to become induced pluripotent stem cells. Int J Nanomedicine 8, 4641–4658 (2013). 24348039 10.2147/IJN.S53830 PMC3857166

[101] Jurlina S. L. A Tet-Inducible CRISPR Platform for High-Fidelity Editing of Human Pluripotent Stem Cells. Genes (Basel) 13, 2363 (2022). 36553630 10.3390/genes13122363 PMC9777998

[102] Agarwal D. Human retinal ganglion cell neurons generated by synchronous BMP inhibition and transcription factor mediated reprogramming. npj Regen Med 8, 55 (2023). 37773257 10.1038/s41536-023-00327-x PMC10541876

[103] Subramanian A. Gene set enrichment analysis: a knowledge-based approach for interpreting genome-wide expression profiles. Proc Natl Acad Sci U S A 102, 15545–15550 (2005). 16199517 10.1073/pnas.0506580102 PMC1239896

[104] Gene Ontology Consortium. The Gene Ontology (GO) database and informatics resource. Nucleic Acids Research 32, D258–D261 (2004). 14681407 10.1093/nar/gkh036 PMC308770

[105] Agrawal A. WikiPathways 2024: next generation pathway database. Nucleic Acids Research 52, D679–D689 (2024). 37941138 10.1093/nar/gkad960 PMC10767877

[106] Milacic M. The Reactome Pathway Knowledgebase 2024. Nucleic Acids Research 52, D672–D678 (2024). 37941124 10.1093/nar/gkad1025 PMC10767911

[107] Liberzon A. The Molecular Signatures Database (MSigDB) hallmark gene set collection. Cell Syst 1, 417–425 (2015). 26771021 10.1016/j.cels.2015.12.004 PMC4707969

[108] Bonilha V. L. Loss of DJ-1 elicits retinal abnormalities, visual dysfunction, and increased oxidative stress in mice. Experimental Eye Research 139, 22–36 (2015). 26215528 10.1016/j.exer.2015.07.014 PMC4573318

[109] Bligh E. G. & Dyer W. J. A rapid method of total lipid extraction and purification. Can J Biochem Physiol 37, 911–917 (1959). 13671378 10.1139/o59-099

[110] Wong A. M. & Budin I. Organelle-Targeted Laurdans Measure Heterogeneity in Subcellular Membranes and Their Responses to Saturated Lipid Stress. ACS Chem. Biol. 19, 1773–1785 (2024). 39069657 10.1021/acschembio.4c00249 PMC11670155

[111] Williamson C. D., Guardia C. M., De Pace R., Bonifacino J. S. & Saric A. Measurement of Lysosome Positioning by Shell Analysis and Line Scan. Methods Mol Biol 2473, 285–306 (2022). 35819772 10.1007/978-1-0716-2209-4_19 PMC11072972

