# Supplementary material for: Age-Driven Lipid Remodeling Activates Lysosome-Mediated Plasma Membrane Repair
Source: Res Sq. 2026 Jan 20:rs.3.rs-8607320. Preprint. [Version 1] doi: 10.21203/rs.3.rs-8607320/v1 (PMC12869690; doi:10.21203/rs.3.rs-8607320/v1)
Supplement: Supplement 1 — Fig. S1. Two-photon fluorescence lifetime imaging reveals sub-retinal deposits in Elovl2C234W mice. a. Relative abundances of LC- and VLC-PUFAs in eyecups from 18-month-old wild-type mice relative to 3-month-old wild-type mice (n = 5 per group). b. Representative in vivo two-photon fundus images from wild-type and Elovl2C234W mice obtained with 760 nm excitation. Top: grayscale intensity images. Middle: FLIM semicircle phasor plots. Clusters of phasor points are color mapped from blue to red, such that red represents highest pixel density. Bottom: FLIM phasor images based on colors assigned in corresponding semicircle phasor plots. c. Representative grayscale two-photon autofluorescence images acquired across 400-660 nm, 400-550 nm, and 580-680 nm wavelength ranges. Statistical significance was determined using unpaired t-tests (a). *P < 0.05, **P < 0.01, ***P < 0.001, and ****P < 0.0001. Source numerical data are available in Source Data. Fig S2. Disrupting LC-PUFA synthesis drives ultrastructural defects and lipid remodeling. a. Relative abundances of LC- and VLC-PUFAs in eyecups from 18-month-old Elovl2C234W mice relative to age-matched wild-type mice (n = 3 per group). b-c. Heatmap (b) and relative abundances (c) of lipid classes in eyecups from 18-month-old Elovl2C234W mice relative to age-matched wild-type (n = 2 per group). Statistical significance was determined using unpaired t-tests (a, b, c). *P < 0.05, **P < 0.01, ***P < 0.001, and ****P < 0.0001. Source numerical data are available in Source Data. Fig S3. ELOVL2 loss remodels plasma membrane lipidome and induces membrane stress. a. qPCR analysis of RPE65 and ELOVL2 expression in differentiated control and ELOVL2KD cells (n = 4 per group). The dashed line indicates expression level in undifferentiated ARPE-19 cells. Data are presented as mean ± SEM. b-c. Relative abundances of LC- and VLC-PUFAs (b) and lipid classes (c) in ELOVL2KD cells compared to control cells (n = 4 per group). d. Immunoblot analysis [file NIHPPrs8607320v1-supplement-1.pdf]

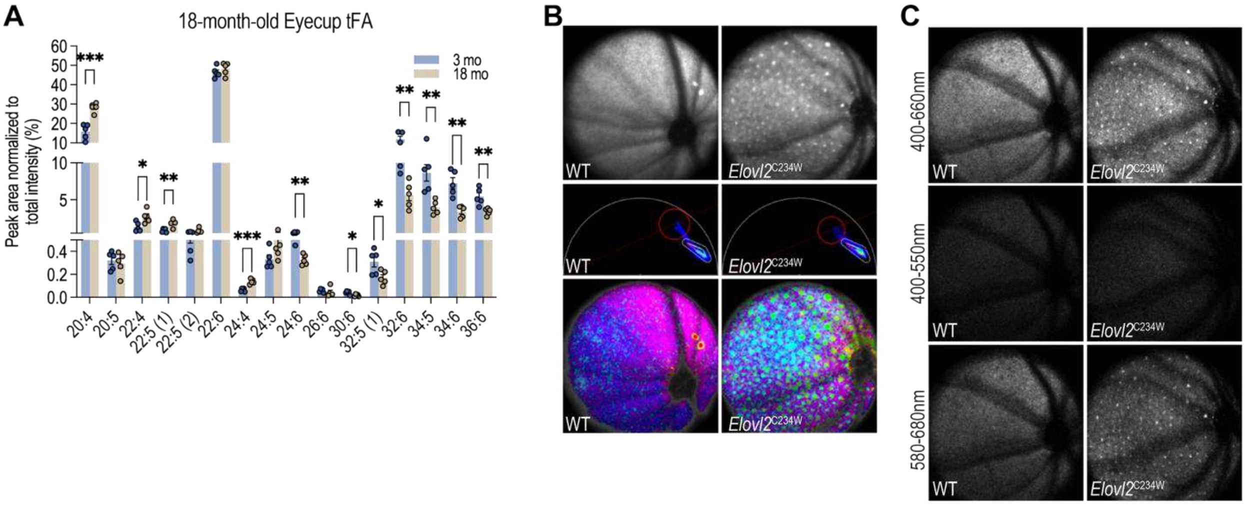
A
18-month-old Eyecup tFA
3 mo
18 mo
Peak area normalized to total intensity (%)
20:4
20:5
22:4
22:5 (1)
22:5 (2)
22:6
24:4
24:5
24:6
26:6
30:6
32:5 (1)
32:6
34:5
34:6
36:6
B
WT
Elovl2C234W
C
400-660nm
400-550nm
580-680nm

Fig. S1

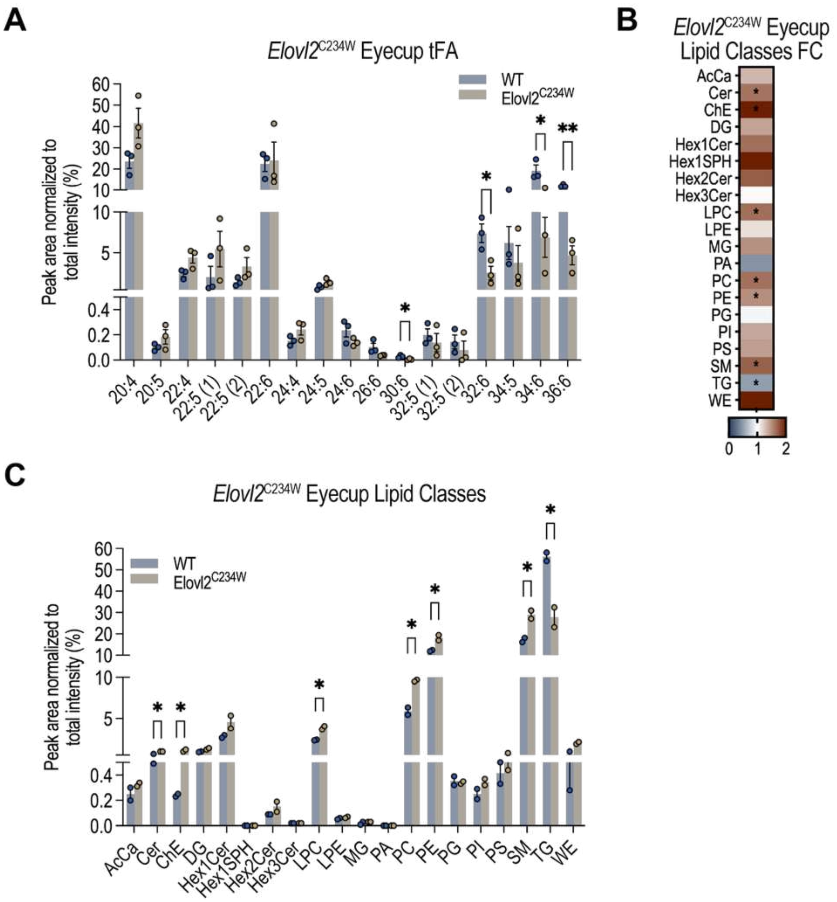
A
Elovl2C234W Eyecup tFA
WT
Elovl2C234W
Peak area normalized to total intensity (%)
20:4
20:5
22:4
22:5 (1)
22:5 (2)
22:6
24:4
24:5
24:6
26:6
30:6
32:5 (1)
32:5 (2)
32:6
34:5
34:6
36:6
B
Elovl2C234W Eyecup Lipid Classes FC
AcCa
Cer
ChE
DG
Hex1Cer
Hex1SPH
Hex2Cer
Hex3Cer
LPC
LPE
MG
PA
PC
PE
PG
PI
PS
SM
TG
WE
0 1 2
C
Elovl2C234W Eyecup Lipid Classes
WT
Elovl2C234W
Peak area normalized to total intensity (%)
AcCa
Cer
ChE
DG
Hex1Cer
Hex1SPH
Hex2Cer
Hex3Cer
LPC
LPE
MG
PA
PC
PE
PG
PI
PS
SM
TG
WE

Fig. S2.

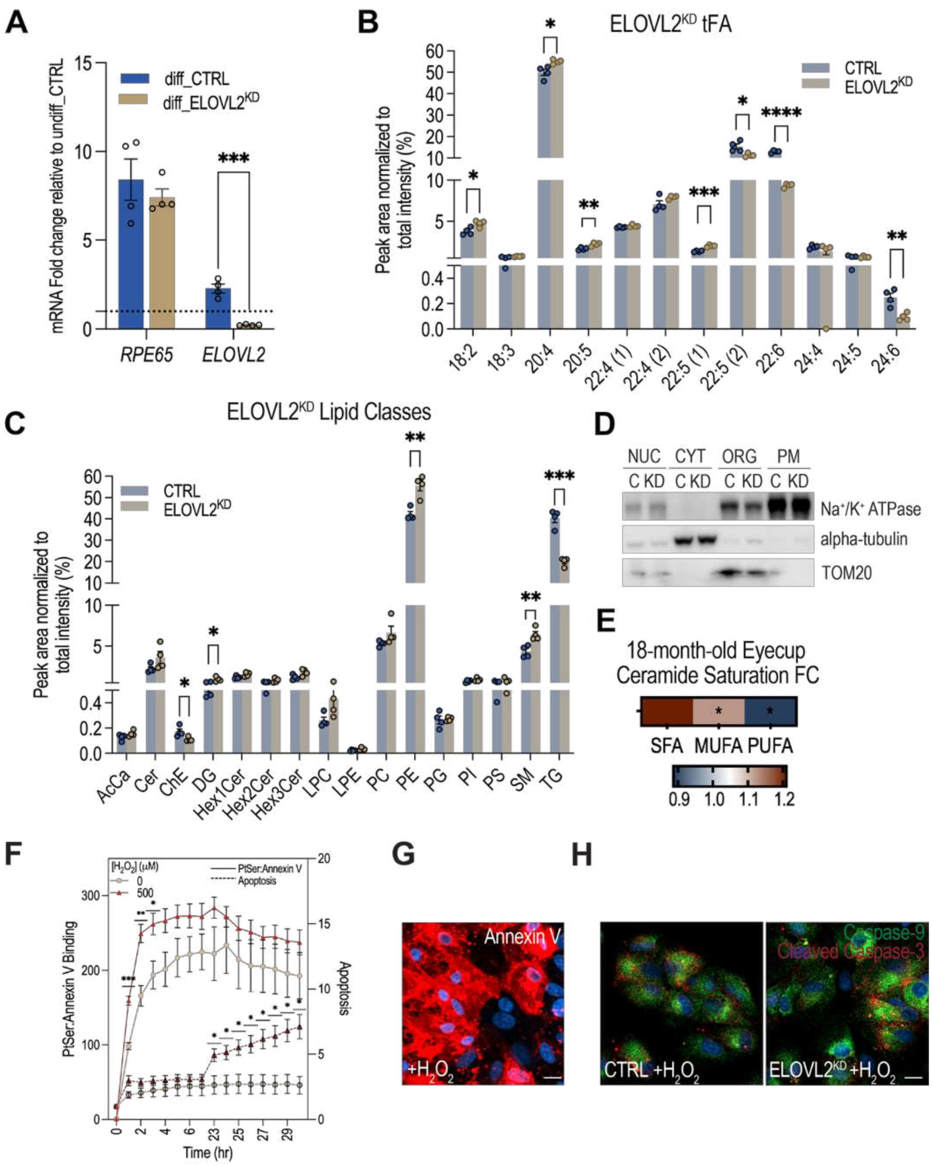
A
mRNA Fold change relative to undiff_CTRL
diff_CTRL
diff_ELOVL2KD
***
RPE65
ELOVL2
B
ELOVL2KD tFA
CTRL
ELOVL2KD
Peak area normalized to total intensity (%)
18:2
18:3
20:4
20:5
22:4 (1)
22:4 (2)
22:5 (1)
22:5 (2)
22:6
24:4
24:5
24:6
C
ELOVL2KD Lipid Classes
CTRL
ELOVL2KD
Peak area normalized to total intensity (%)
AcCa
Cer
ChE
DG
Hex1Cer
Hex2Cer
Hex3Cer
LPC
LPE
PC
PE
PG
PI
PS
SM
TG
D
NUC
CYT
ORG
PM
C KD C KD C KD C KD
Na+/K+ ATPase
alpha-tubulin
TOM20
E
18-month-old Eyecup
Ceramide Saturation FC
SFA
MUFA
PUFA
0.9
1.0
1.1
1.2
F
[H2O2] (uM)
0
500
PtSer:Annexin V
Apoptosis
PtSer:Annexin V Binding
Apoptosis
Time (hr)
G
Annexin V
+H2O2
H
Caspase-9
Cleaved Caspase-3
CTRL +H2O2
ELOVL2KD +H2O2

Fig. S3.

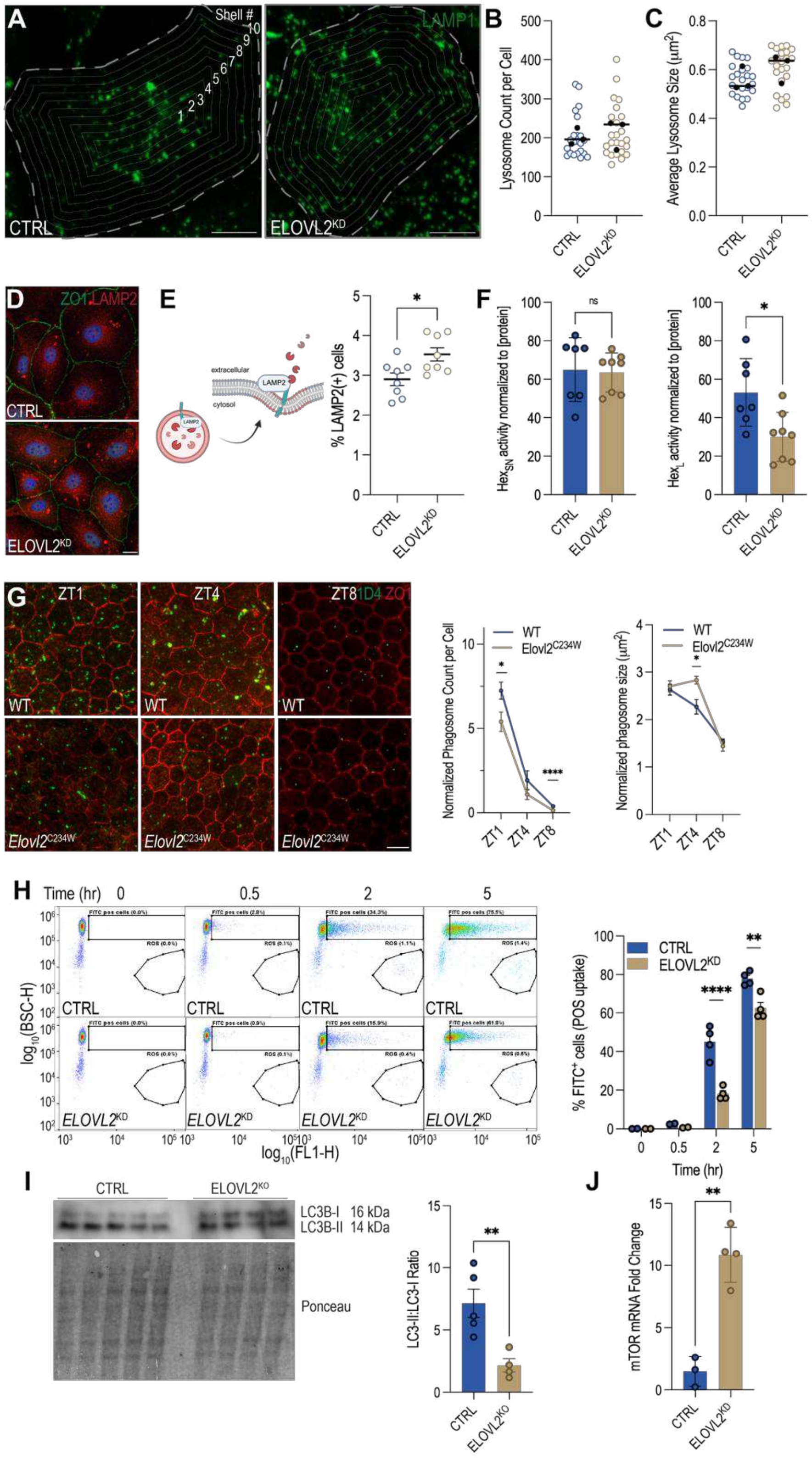
A
Shell #
1 2 3 4 5 6 7 8 9 10
LAMP1
CTRL
ELOVL2KD
B
Lysosome Count per Cell
CTRL
ELOVL2KD
C
Average Lysosome Size (μm2)
D
ZO1 LAMP2
E
extracellular
cytosol
LAMP2
% LAMP2(+) cells
F
HexSN activity normalized to [protein]
HexL activity normalized to [protein]
ns
G
ZT1
ZT4
ZT8 1D4 ZO1
WT
Elovl2C234W
Normalized Phagosome Count per Cell
Normalized phagosome size (μm2)
H
Time (hr)
0
0.5
2
5
log10(BSC-H)
log10(FL1-H)
FITC pos cells
ROS
% FITC+ cells (POS uptake)
I
ELOVL2KO
LC3B-I 16 kDa
LC3B-II 14 kDa
Ponceau
LC3-II:LC3-I Ratio
J
mTOR mRNA Fold Change

Fig. S4.

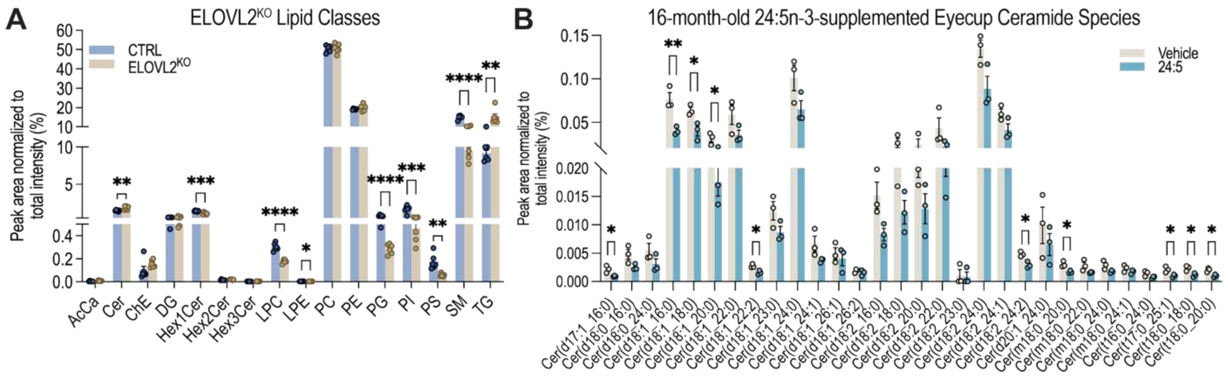
A
ELOVL2KO Lipid Classes
CTRL
ELOVL2KO
Peak area normalized to total intensity (%)
AcCa
Cer
ChE
DG
Hex1Cer
Hex2Cer
Hex3Cer
LPC
LPE
PC
PE
PG
PI
PS
SM
TG
B
16-month-old 24:5n-3-supplemented Eyecup Ceramide Species
Vehicle
24:5
Peak area normalized to total intensity (%)

Fig. S5

Antibody/Probe Name	Species	Dilution	Company (Catalog #)	RRID
phalloidin	-	1:40	Molecular Probes (F432)	-
ZO-1	rabbit	1:100	Invitrogen (61-7300)	AB_2533938
mCLING	-	500 nM	Synaptic Systems (710 006AT647N)	-
Ceramide	mouse	1:100	Glycobiotech (MAB_0014)	-
Annexin V	-	1:40	Invitrogen (A35108)	-
Caspase-9	mouse	1:100	Santa Cruz Biotechnology (56076)	AB_781847
Cleaved caspase-3	rabbit	1:100	Cell Signaling (9664)	AB_2070042
LAMP-1	rabbit	1:100	Invitrogen (MA5-29385)	AB_2785256
LysoTracker	-	50 nM	Invitrogen (L7526)	-
CellMask	-	1:1000	Invitrogen (C10046)	-
LAMP-2	mouse	1:100	Santa Cruz Biotechnology (18822)	AB_626858
1D4	mouse	1:500	Produced in-house from hybridoma cells	-
LC3B	rabbit	1:1000	Sigma-Aldrich (L7543)	AB_796155
APOE	goat	1:100	MilliporeSigma (AB947)	AB_2258475
LAMP-1	rabbit	1:100	Cell Signaling (99437)	AB_3065089
Hoechst 33342	-	1:2000	Invitrogen (H1399)	-

Supplementary Table 1. List of antibodies and probes used in this study.

Gene	Sequence	Company
Human *ELOVL2* siRNA	Sense strand 5'-GGCUACAACUUACAGUGUCAAGATC-3' and Anti-sense strand 5'-GAUCUUGACACUGUAAGUUGUAGCCUC-3';	Integrated DNA Technologies

Supplementary Table 2. List of siRNA sequences used in this study.

Gene	Sequence	Company
Human *ELOVL2*	forward 5'-CTTGGGAAGGAGGCTACAAC-3' and reverse 5'-CACCAAAGCACCTTGGCTAC-3';	Integrated DNA Technologies
Human *RPE65*	forward 5'-AAAAATGCCAGAAAGGCTCC-3' and reverse 5'-CAGAATTGCAGTGGCAGTTG-3';	Integrated DNA Technologies
Human *TBP*	forward 5'-TGTATCCACAGTGAATCTTGGTT -3' and reverse 5'-GGTTCGTGGCTCTCTTATCCTC-3';	Integrated DNA Technologies
Human *CERS2*	forward 5'-TTCTGGTGGGAACGTCTGTG-3' and reverse 5'-CTTTGGCGTAGACACGTCCAT-3';	Integrated DNA Technologies
Human *mTOR*	forward 5'- ATGCTTGGAACCGGACCTG-3' and reverse 5'- TCTTGACTCATCTCTCGGAGTT-3';	Integrated DNA Technologies

Supplementary Table 3. List of primer sequences used for real-time quantitative PCR (qPCR) in this study.
